# Optimizing the fracture resistance of clay liners through fiber content and moisture control

**DOI:** 10.1038/s41598-025-17178-9

**Published:** 2025-09-12

**Authors:** Mohammad Zaid, Zarghaam Rizvi, Dipanjan Basu, Frank Wuttke

**Affiliations:** 1 Department of Civil and Environmental Engineering, University of Waterloo, Waterloo, Canada; 2 Geomechanics and Geotechnics, University of Kiel, Kiel, Germany; 3 GeoAnalysis Engineering, Kiel, Germany

**Keywords:** Fracture toughness, Clayey soil, Landfill liners, Glass fiber, Optimum moisture content, Crack resistance, Environmental sciences, Engineering

## Abstract

Structural integrity of clay liners in engineered waste landfills depends critically on their ability to resist the initiation and propagation of cracks under variable moisture conditions. In this study, the improvement of Mode I Fracture toughness K_I_ in clayey soil through small additions of discrete glass fibers is investigated with particular emphasis on the interaction between fiber content and water content near the optimum moisture content (OMC). Specimens were prepared using a clayey soil compacted at water contents of 17%, 18%, and 19%, representing dry, optimum, and wet of optimum states based on proctor compaction test. Glass fibers were added uniformly at fractions of 0%, 0.01%, 0.02%, 0.05%, and 0.10% by weight of the soil. K_I_ was obtained from single-edge notched beam (SENB) specimens tested in a three-point bending configuration. Load and displacement responses were analyzed to extract peak load P_max_ and compute K_I_. The addition of only 0.01% glass fiber by mass enhances P_max_ by 50%, resulting in a 70% increase in K_I_ across all moisture conditions. These improvements are attributed to the effective interplay between clay particles bonded together with the glass fibers. The results also indicate that both P_max_ and K_I_ reach their maximum values near OMC (~ 18%), corresponding to the densest particle arrangement. However, increasing the fiber content beyond 0.01% leads to a decrease in K_I_ and P_max_ caused by fiber clustering, void formation, and weakened soil-fiber interfaces. The findings clearly illustrate that, by precisely limiting the water content and adding a sub-percent amount of glass fiber reinforcement, fracture resistance in clay liners increases significantly. This state-of-the-art approach offers a cost-effective and technically efficient strategy for enhancing the long-term performance of landfill systems to prevent seepage of harmful leachate to the groundwater.

## Introduction

Landfills are critical engineering structures used for waste management in modern-day cities and date back to the early 20th century. Landfills are primarily designed and constructed based on their specific functions and are categorized as: (a) Municipal Solid Waste (MSW) landfills, (b) Industrial Waste landfills, (c) Hazardous Waste landfills, (d) Construction and Demolition (C&D) landfills, and (e) Bioreactor landfills^1–3^. These landfills have six critical parts: (a) bottom liner system, (b) waste cells, (c) leachate collection system, (d) gas collection system, (e) cover system, and (f) stormwater drainage system^1,3,4^. Among these, the bottom liners are made from clay or clayey soils that serve as essential barriers for preventing harmful substances found in landfills, such as per- and poly-fluoroalkyl substances (PFAS), often referred to as forever chemicals, from leaching into the groundwater. PFAS refers to a category of synthetic chemicals used in a wide range of everyday products^5^. PFAS are recognized for their ability to withstand heat, grease, and moisture. Due to their extensive use and durability, PFAS can be detected in air, water, soil, and the bloodstream of humans^5^. Although research on their health impacts is ongoing, certain PFAS have been associated with possible health hazards. When a clay liner develops cracks, generating preferential flow paths, the effective permeability of the clay liner increases from its designed value. Consequently, groundwater gets contaminated with PFAS and the clay liner fails to perform its intended function.

Over the years, several incidents related to the failure of landfill clay liners have been reported in North America. The Kettleman Hills landfill in California experienced leakage in the 1980s due to desiccation cracks in its clay liner. Inadequate liner systems in Love Canal, New York resulted in toxic chemical contamination in the surrounding communities. Concerns about hazardous waste leaking into groundwater emerged in the 1990s for the Emelle landfill in Alabama due to reported liner issues. Clay liner shrinkage during dry conditions led to cracking and increased movement of leachate in the southside Landfill in Indiana. The Halton landfill in Ontario faced performance challenges in the 1990s, resulting from insufficient clay compaction, which raised concerns about seepage. Several reports from the early 2000s highlighted leachate leakage in Richmond landfill in Napanee, Ontario, raising questions regarding the effectiveness of the clay liner. The Cache Creek landfill in British Columbia experienced operational difficulties, which highlighted the clay liner’s vulnerability to freezing and thawing cycles. Planning and construction of the Stoney Creek landfill in Hamilton, Ontario, faced substantial public opposition in the late 1980s and early 1990s over concerns regarding liner degradation and the potential for contamination of nearby water sources^6–22^. These cases clearly show that, while clay liners are commonly used, these can fail due to several critical factors such as (a) drying and cracking, (b) incorrect installation or compaction, (c) chemical deterioration, (d) varying settlements, and (e) climate-related stresses, like freeze-thaw cycles. Among these, differential settlement is an important issue because it can lead to cracks in clay liners, and this has not been investigated from a fracture mechanics point of view.

Many landfill liner failures in Canada and the United States underscore the importance of understanding how clayey soils cracks^5,8,16,17,23^. Cases like the Kettleman Hills landfill in California and the Richmond landfill in Ontario demonstrate that cracks can significantly compromise the integrity of clay liners^8,24^. These cracks can occur due to drying out, mechanical stress, or freeze-thaw cycles. Such cracks increase the flow of liquid through the liners, allowing harmful leachate to escape into the surrounding area, which can lead to significant environmental and operational issues. Although clay soils are commonly used in liner systems and have been shown to work well under various loads in laboratory and field studies, traditional tests often incorrectly assume a uniform, flawless material. It is known that clay soils can develop small and large cracks, especially between certain moisture levels and even below the optimum moisture condition. These natural flaws challenge the idea of uniform behaviour and require a closer look at how clayey soils crack. Clayey soils are widely used in infrastructure applications such as pavement sub-layers, pipeline bedding and backfill, and embankment dams. Consequently, their mechanical properties critically influence both the immediate and long-term performance of these systems. Prior studies have largely focused on general physical and mechanical parameters—tensile and shear strength, frictional behavior, microstructure, workability, shrinkage, plasticity, permeability, and settlement^25–29^. However, soils are susceptible to deformation and cracking due to their inherently low tensile strength, particularly under tensile or shear loading. These deficiencies can lead to interlayer cracking, excessive settlement, and swelling. Therefore, understanding the fracture and cracking resistance of soils is important^30–33^. By better understanding how and when these cracks initiate, grow, and affect fluid movement, engineers can more accurately predict liner performance and create stronger waste containment systems.

**Fig. 1:**
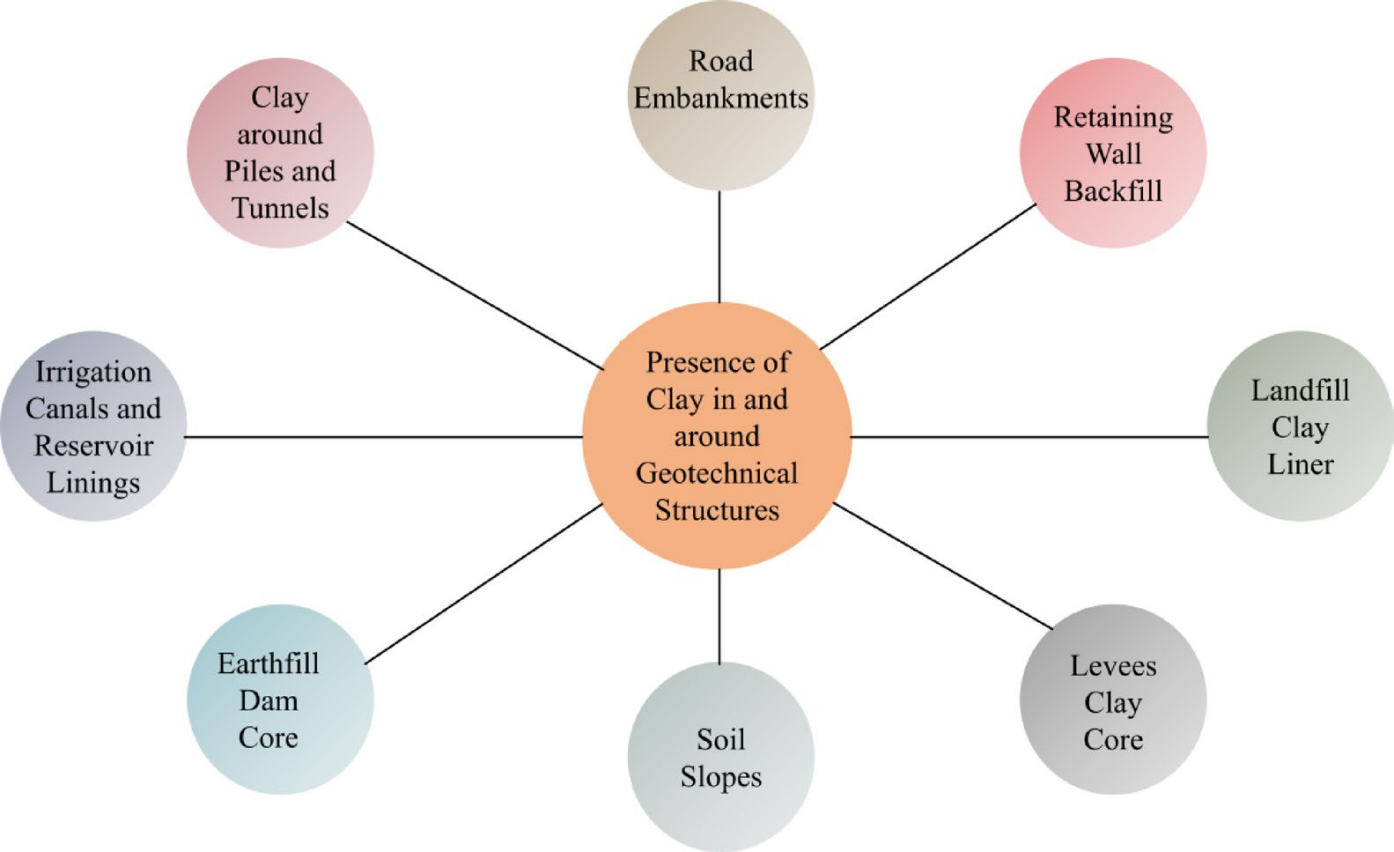
Impact of clayey soil on the stability of various geotechnical structures.

**Fig. 2:**
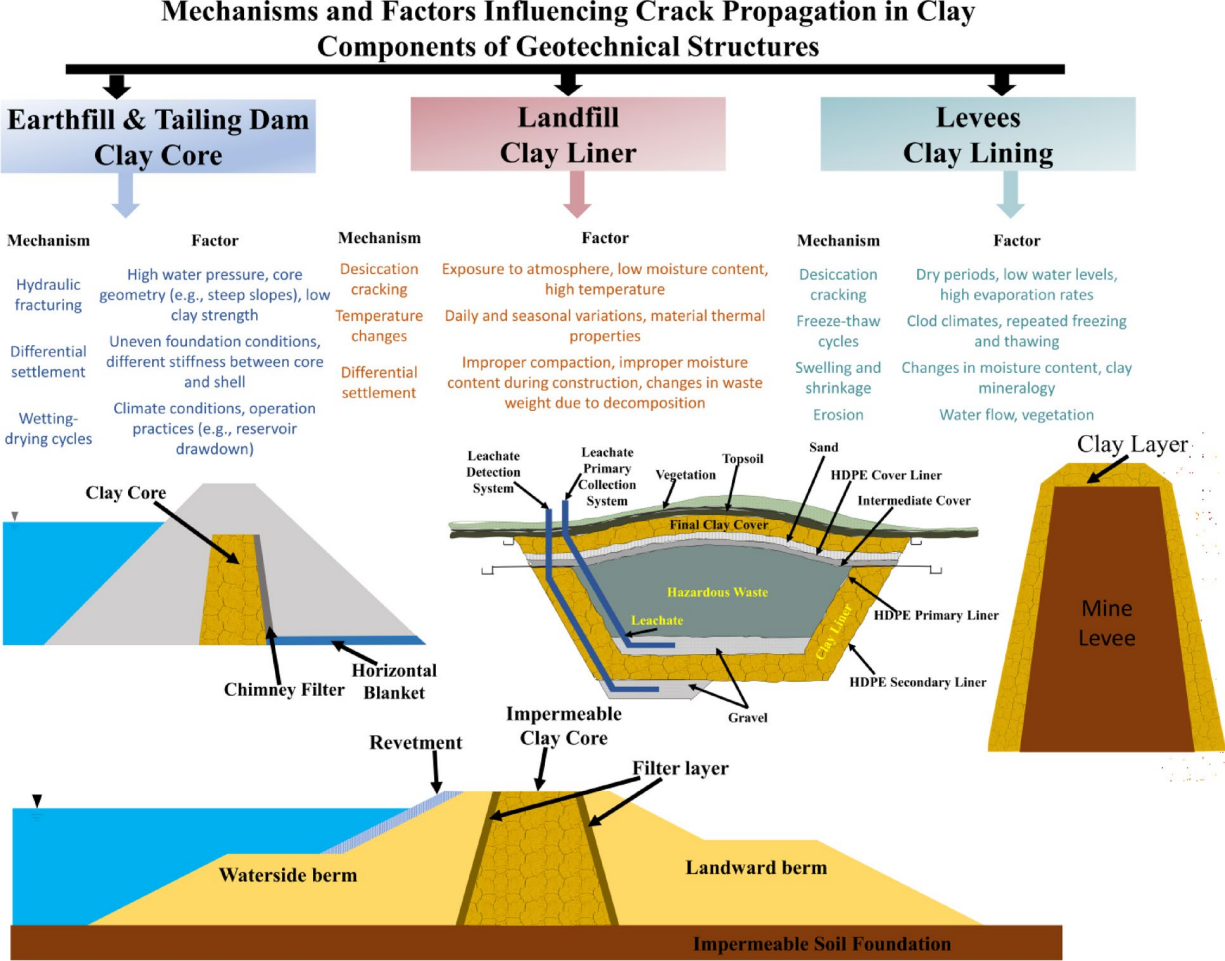
Components of geotechnical structures composed of clayey soils where cracks develop.

Fractures in clayey soils caused by deformation due to differential settlement can also occur in various other geostructures. Figure 1 shows different geotechnical structures that can be impacted detrimentally by the presence of clayey soils. Figure 2 presents various mechanisms and associated factors that lead to crack formation in clayey soil components of four major engineering structures. Understanding tensile fracture behaviour in clay remains particularly relevant to applications such as hydraulic fracturing, desiccation cracking, differential settlement, and slope failure^34,35^. Clayey soils are formed through prolonged weathering of silicate-rich rocks by both physical disintegration and chemical alteration that reduce mineral particles to sizes smaller than 2 microns^36,37^. The fine-grained characteristics impart distinct mechanical and hydraulic properties to clays. The primary clay minerals kaolinite, illite, and montmorillonite are phyllosilicates characterized by layered structures and composed of silicon-oxygen tetrahedra and aluminum-oxygen/hydroxyl octahedra^38,39^. The arrangement of these sheets influences behaviours such as swelling, shrinkage, and water retention. Water within these soils exists in various forms, including adsorbed, interlayer, and capillary forms, each of which affects the soil’s strength and deformation properties^40,41^. Mechanically, normally consolidated (NC) clays tend to exhibit contractive behaviour under shear stress due to their particle size and sensitivity to water^41^. The Critical State Soil Mechanics (CSSM) framework accurately characterizes the shear deformation and volumetric change of clay behaviour^42–44^. The fracture behaviour of clays is analyzed using Linear Elastic Fracture Mechanics (LEFM), a method developed for brittle materials. LEFM focuses on the growth of existing cracks under elastic conditions, defined by the stress intensity factor. It states that fractures occur when the stress intensity at the crack tip reaches a critical level, known as fracture toughness, typically without significant plastic deformation^45^. LEFM identifies three types of crack propagation: Modes I, II, and III. Mode I, characterized by tensile opening, is particularly significant for clays, especially during desiccation or when subjected to tensile stress. While the concepts of fracture mechanics initially emerged from the study of metals and structural materials, these concepts are now being increasingly used in geotechnical applications to better understand crack initiation and growth in clayey soils^34,46–49^. LEFM method facilitates more accurate predictions of failure mechanisms in clay liners and various other geotechnical systems.

To effectively characterize a soil’s resistance to crack propagation, several key fracture parameters are considered such as fracture toughness (K_I_), crack tip opening displacement (CTOD), and energy release rate (G_IC_)^34,50–54^. Among these, K_I_ stands out as the most critical parameter defining the essential stress intensity factor required for crack propagation. It serves as a definitive measure of a soil’s capacity to resist crack initiation and growth, which is vital in maintaining the structural integrity of compacted clay liners.

K_I_ for mentals and alloys can be accurately measured using various methods, such as the Single Edge Notched Tension (SENT), Single Edge Notched Beam (SENB), Center Cracked Tension (CCT), Double Edge Notched Tension (DENT), and Compact Tension (CT)^45^. Notably, the SENB method is acclaimed as the most reliable choice for clayey soils. The method provides a clear and standardized approach to applying controlled loading while effectively capturing crack propagation behaviour under Mode I conditions. The SENB technique minimizes uncertainties related to specimen preparation and loading geometry, positioning it as an optimal method for scientific research and quality control in geotechnical applications^55–58^.

The LEFM theory was developed for metals and alloys, and several tests were performed to establish standard testing procedures and propose standardized equations for the estimation of fracture toughness of materials^45,58–60^. The LEFM theory was also applied to rocks for estimation of K_I_ under static and dynamic loading conditions to investigate the behaviour of rocks under hydraulic fracturing^61–69^. These studies focused on the intrinsic and extrinsic properties of rocks and relied heavily on the orientation of stratification layers and other existing failure planes^70–74^. These studies considered both the SENB and Semi-Circular Bend Notch specimens. Numerical methods such as the discrete lattice element method have been applied to study fracture initiation and growth in geomaterials^75^. Further, coupled effects of temperature and moisture variations on the crack development processes in soil have been investigated by numerically and experimentally^76,77^. Notwithstanding, studies on the determination of K_I_ for soils are relatively rare.

No international test procedure has yet been proposed to determine the static and cyclic crack resistance characteristics of soils and soft geomaterials under pure fracture modes I, II, and III or under general combined failure modes I/II and I/III. SENB fracturing (i.e., Mode I) is the failure mode that several scholars have recently investigated due to the weakness of geomaterials under tension^78,79^. In fact, fracture Mode I is the most frequently encountered mode in landfill clay liners, dam clay cores and clay cores of mine tailings, among the three basic fracture modes^80^. Therefore, it is important to develop convenient testing approaches for accurately measuring K_I_ of geomaterials. The International Society for Rock Mechanics and Rock Engineering (ISRM) has suggested several standard testing methods for determination of K_I_ of rock materials^81^. However, clayey soils, which are often used as cores of dams and liners in landfills, have been investigated less frequently than rocks. K_I_ of clay soils has been investigated by different researchers^34,49,50^^82^–^86^. Amarasiri et al.,^34^ noted that it is difficult to conduct tests on large-sized SENB specimens of clayey soils because the specimens have low strength and self-weight impacts the results. Wang et al.,86 proposed an improved method in which the effect of self-weight is eliminated. Wang et al.,^49^ experimentally determined K_I_ of compacted clay using NSCB specimens. They investigated the effects of notch length, thickness, moisture content, and dry density on K_I_ and recommended a range of 0.3 to 0.5 for a/R for the determination of K_I_ of compacted clays (a = crack length and R = radius of specimen). No significant thickness-dependent size effect was observed in their experiments. Based on SCB testing, they proposed an empirical relationship K_I_ = 0.3283σ_t_ connecting K_I_ and tensile strength (σ_t_) of directly compacted clays. The NSCB method proved efficient and effective for fracture testing in compacted clays. Erarslan & Aliha^87^ employed the chevron notched semicircular bend (CN-SCB) method to characterize K_I_ in cement-stabilized sandy clay samples subjected to static and cyclic loading. Their study, which included both fine and coarse-grained soils with low (2%) and high (10%) cement contents, demonstrated that K_I_ increases with increase in grain size and cement content under static conditions, reaching a maximum value of 0.235 MPa·m^0^^5^. However, cyclic loading reduces K_I_ by approximately 25%, with the coarse-grained soils exhibiting a greater degradation than fine-grained soils due to enhanced intergranular cracking. The fine-grained soils, in contrast, displayed improved fatigue life, a reflection of their higher ductility. These experimental insights were supported by numerical simulations, which affirmed the roles of cement content, gradation, and loading regime in governing fracture behaviour. The semicircular bending (SCB) test has become widely adopted in soil mechanics because of its ability to effectively model both mode I and mixed-mode fractures. Studies by Wang et al.,^49^, Zhang et al.,^51,88^, and Song et al.,^89^ illustrate the adaptability of the SCB approach in investigating the effects of variables such as crack length, specimen thickness, moisture content, and additives (e.g., fly ash) on fracture performance in compacted clay and mine tailings. Notably, Song et al.,^89^ linked increases in cement content and curing time while Qiao et al.,^90^ and Rodriguez et al.,^91^ demonstrated the sensitivity of K_I_ to dry density, water content, and suction effects, particularly for bentonite clays. Aliha et al.,^92^ introduced an asymmetric SCB (ASCB) specimen to capture mixed-mode I/II fracture behaviour in compacted clay. Using the maximum tangential stress criterion, they found K_I_ values between 0.025 and 0.035 MPa·m^0^^5^, with K_I_ reduced by 30% under combined tensile and shear loading. This configuration provides a practical tool for characterizing the fracture response in subgrade soils and embankments where mixed-mode conditions prevail.

Various test methods have been developed to quantify K_IC_ in soils, including compact tension (CT), SENB, indirect tensile, cracked ring, and Brazilian disc configurations^82,84,86^^93^–^95^. Among these, SCB and SENB tests have become standard due to their simplicity and ability to incorporate environmental variables such as moisture and density^96,97^. Hanson et al.,^98^ highlighted the inverse relationship between water content and K_IC_ for CL and ML soils, consistent across multiple bending configurations^99^. Recent innovations include the use of Digital Image Correlation (DIC) to quantify displacement fields and crack tip parameters in clay specimens. Qiao et al.,^90^ applied DIC to notched SCB specimens of Gaomiaozi bentonite and demonstrated that the K_IC_ fracture process zone (FPZ) length and critical crack tip opening displacement peaked at optimal moisture content (~ 9.5%) and increased with dry density. The coupled role of moisture and compaction is also evident in red clay systems studied by Ma et al.,^100^where gravel content and saturation levels influenced cracking through incubation, propagation, and final fracture stages. In addition to experimental investigations, optimization techniques such as genetic algorithms (GA) and particle swarm optimization (PSO) have been widely applied in geotechnical engineering to model complex soil behaviours and design parameters^101^.

Early foundational work by Lee et al.,^102^ introduced compact tension testing of overconsolidated clays, emphasizing the role of critical energy release rate (G_c_) as a material constant to define tension cracking in soils. Later, Nishimura & Shimuzu^103^ demonstrated that the ratio of crack length and specimen size is essential in keeping K_I_ values consistent. More recent studies^49,50,82^ expanded these insights to frozen and unsaturated soils, incorporating empirical relationships and nonlinear fracture models. Red clay, a commonly used fill material available in tropical and subtropical regions, presents unique challenges related to fracture due to its mineral composition and sensitivity to moisture^104,105^. Even under high compaction, red clay remains susceptible to cracking due to traffic induced cyclic loading or environmental desiccation, thereby undermining infrastructure durability^106,107^. Fracture tests on compacted red clay by Wang et al.,^108^using prefabricated notches and D_IC_ imaging, revealed a nonmonotonic relationship between moisture and K_I_, along with progressive FPZ development extending beyond peak load (P_max_). These observations highlight the quasi-brittle nature of clay and the need to capture the evolving FPZ, which linear elastic models often fail to account for.

The LEFM theoretical framework has become increasingly relevant for soils, despite its original development for brittle materials such as concrete and rock^52,109^. Traditional yield criteria, such as the Mohr-Coulomb criterion, fall short in characterizing crack propagation, particularly in stiff or over-consolidated clays^110^. LEFM-based studies are now frequently used to explore the relationship between K_I_ and tensile strength (σ_t_), with correlations validated across various geomaterials^64,111,112^. Wang et al.,^84^ established a linear relation, K_I_ = 0.3546σ_t_, for compacted clays, offering a simplified estimation method for K_I_ via uniaxial tensile strength measurements. Furthermore, the role of sample geometry, loading rate, and crack tip plasticity in influencing measured K_I_ values continues to challenge standardization^113,114^. Modelling the fracture response of red clay requires greater refinement, especially in accounting for its pronounced heterogeneity and water sensitivity. As geotechnical systems increasingly rely on local soils for critical infrastructure construction, a deeper mechanistic understanding of their fracture behaviour is essential. Integrating empirical observations with robust physical models and standardizing testing procedures across diverse soil types is essential for significantly advancing the predictive capabilities of fracture mechanics in geotechnical engineering. This approach will undoubtedly lead to more reliable and accurate assessments in the field.

Literature lacks studies on clayey soils based on fracture mechanics, such as the estimation of fracture toughness, maximum load capacity and corresponding displacement. Additionally, the presence of additives in clayey soils, such as glass fibers has largely been overlooked in the systematic investigation of how fiber reinforcement can significantly enhance the fracture resistance of landfill clay liners. Recognizing this critical gap, the current study employs glass fiber reinforcement to rigorously explore its impact on improving fracture toughness in clayey soils. The experimental phase begins with solid SENB testing of unreinforced soil specimens across a moisture content range of 16–20%, establishing a clear relationship between water content and fracture toughness. This enables the identification of the optimal moisture condition that yields the maximum K_I_ values. Subsequently, the glass fiber content is varied from 0.01 to 0.10% by mass at three pivotal moisture levels: 17% (below optimum), 18% (at optimum), and 19% (above optimum). Through this comprehensive and meticulously designed testing matrix, the research determines the most effective fiber content that maximizes fracture toughness, thereby substantially improving the structural integrity and overall performance of landfill clay liners. These findings provide practical, evidence-based guidelines that significantly advance the development of more durable and reliable containment systems.

### Soil characterization and properties

This study analyzes five soil samples, as described in Table 1, and performs fracture toughness testing on the most suitable soil sample, which exhibits the lowest hydraulic conductivity. Initially, soil properties have been obtained through laboratory tests which are conducted following standard procedures recommended by the American Society for Testing and Materials (ASTM)^99,115–122^. These tests aim at thoroughly examining the physical, mechanical, and chemical characteristics of the soil samples. The goal is to identify a sample with suitable properties for K_I_ evaluation using the SENB method.

**Fig. 3:**
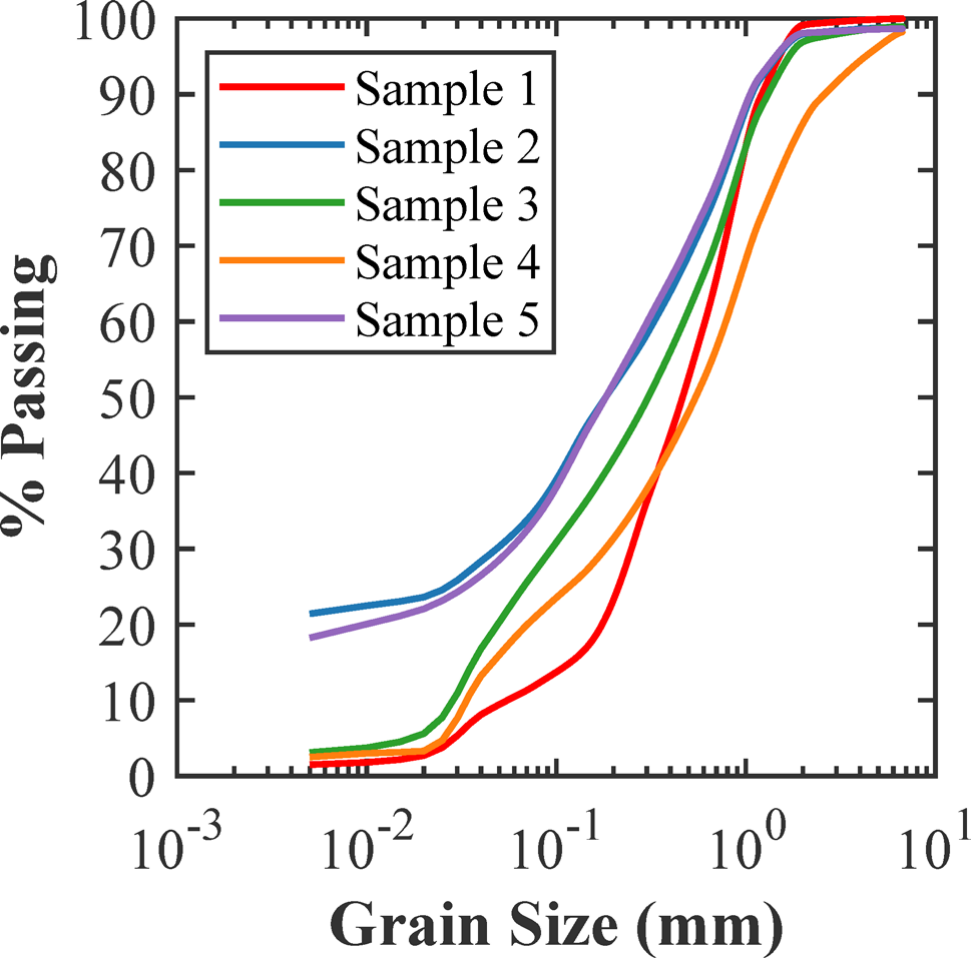
Grain size distribution of the soil samples. .

**Fig. 4:**
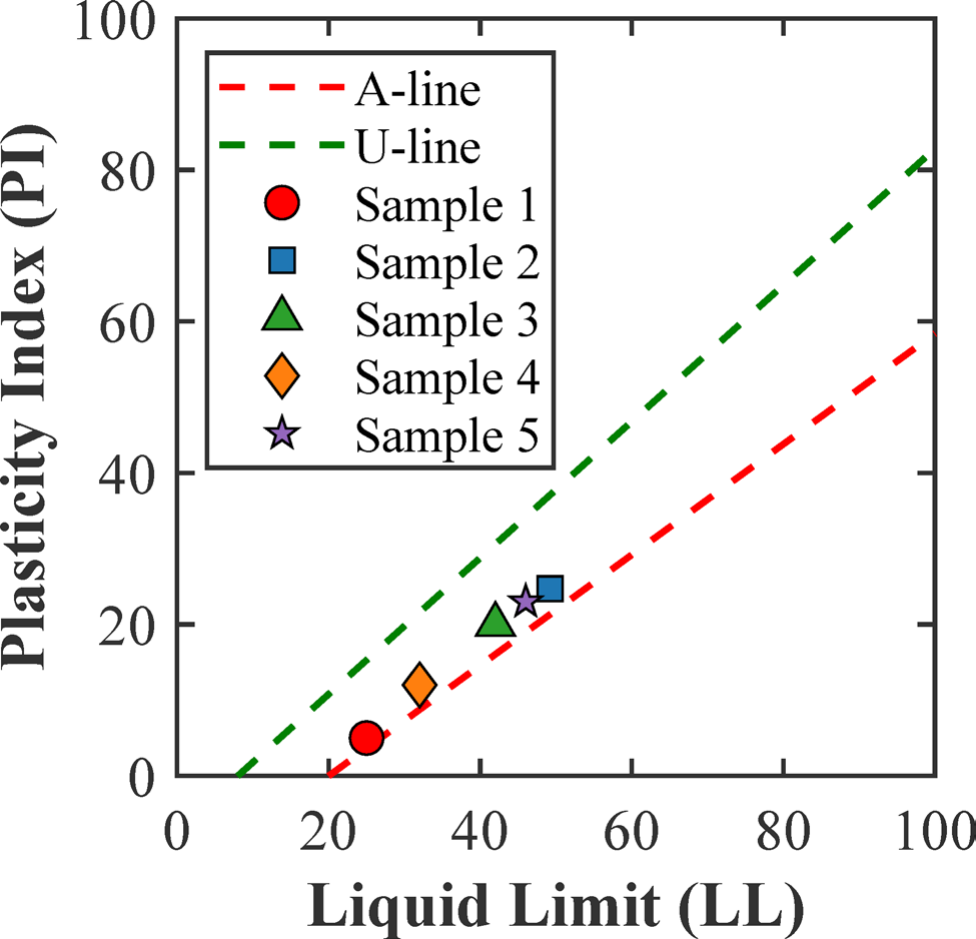
Positions of soil samples in Casagrande’s plasticity chart.

The grain size distributions of the samples, presented in Fig. 3, exhibit a wide range of particle sizes, from clay-sized particles to fine sands. Samples 2 and 5 have a significantly high percentage of fine particles, especially clay fraction (< 0.002 mm). The high fines content implies high plastic properties useful for creating stable, low-permeability samples essential for effective fracture testing. The characteristics of plasticity are evaluated using the Casagrande plasticity chart presented in Fig. 4. Sample 2 plots well above the A-line, categorizing it as a low plasticity clay (CL) according to the Unified Soil Classification System (USCS). Sample 2 has a liquid limit of 49.2% and a plasticity index of 24.7% (Table 1), reflecting a material that can sustain considerable strain before failure. In contrast, other samples, like Sample 1, displayed lower plasticity, classifying them as low plasticity clays (CL) with relatively more brittle behaviour.

**Fig. 5:**
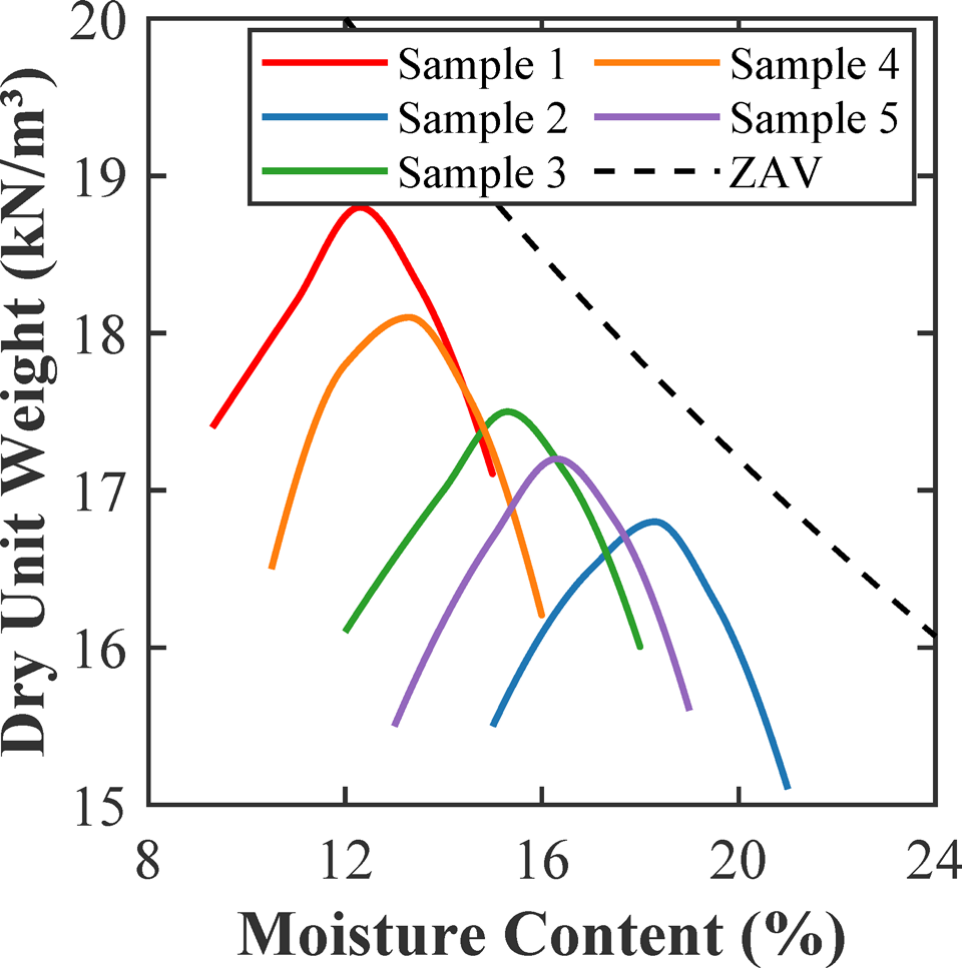
Proctor compaction test result (ZAV = Zero Air Void Line).

The compaction behaviour of each soil sample is examined using Standard Proctor tests, and the results are shown in Fig. 5. Sample 2 reached a maximum dry unit weight of 16.8 kN/m³ at an optimum moisture content (OMC) of 18.3%. This moisture-unit weight relation suggests that the soil sample 2 requires a moderate water content for optimal compaction, which is typical for low plasticity clays. Although its maximum unit weight is slightly lower than that of other samples, Sample 2’s compaction profile demonstrates its ability to create dense, uniform specimens, which is a vital aspect that helps in reducing variability during K_I_ tests. Hydraulic conductivity measurements show that Sample 2 has a very low permeability of 0.235 × 10^− 9^ m/s (Table 1), which reflects a dense and uniform microstructure. Therefore, Sample 2 is the most suitable soil, as it has low permeability, a basic requirement for municipal soil waste landfills to minimize leachate leakage and contaminant migration into the groundwater^23^.

**Table 1:** Summary of soil site investigation results.

*Soil Sample*	G_S_	L.L.	*P*.I.	${\gamma _{d,\hbox{max} }}$(kN/m³)	O.M.C. (%)	k (×10⁻⁹ m/s)	U.S.C.S. Classification
Sample 1	2.66	25	5	18.8	12.3	9.886	CL (Low plasticity clay)
Sample 2	2.71	49.2	24.7	16.8	18.3	0.235	
Sample 3	2.69	42	20	17.5	15.3	1.294	
Sample 4	2.67	32	12	18.1	13.3	5.272	
Sample 5	2.7	46	23	17.2	16.3	0.838	

G_s_ is Specific Gravity, L.L. is Liquid Limit, P.I. is Plasticity Index, $\:{\gamma\:}_{d,max}$ is Maximum Dry Density, O.M.C. is Optimum Moisture Content, k is Hydraulic Conductivity, U.S.C.S. is United Soil Classification System.

**Fig. 6:**
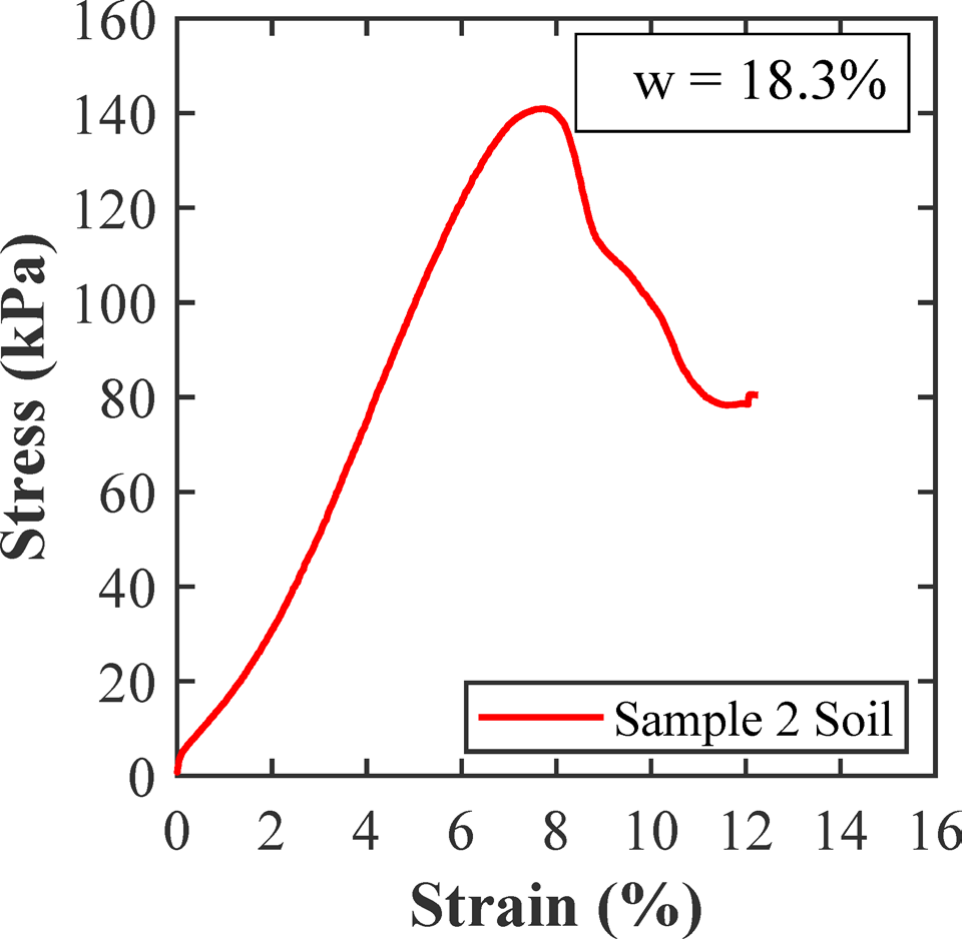
Stress-strain response of soil sample 2 used in the K_I_ test.

Unconfined compressive strength (UCS) test is performed on Sample 2 to evaluate its mechanical response under axial load. The stress-strain curve from the UCS test is shown in Fig. 6. Sample 2 reached a peak stress of 142 kPa at an axial strain of 7.75%. After achieving the peak, the stress gradually diminished as the strain increased, indicating ductile strain-softening behaviour instead of abrupt brittle failure. This capacity for considerable deformation after the peak is crucial for studies on K_I_, emphasizing the importance of stable crack growth and energy absorption mechanisms. Due to its high plasticity, moderate strength, ductile mechanical response, and low permeability, Sample 2 is chosen for additional K_I_ assessment using the SENB sample configuration.

**Fig. 7:**
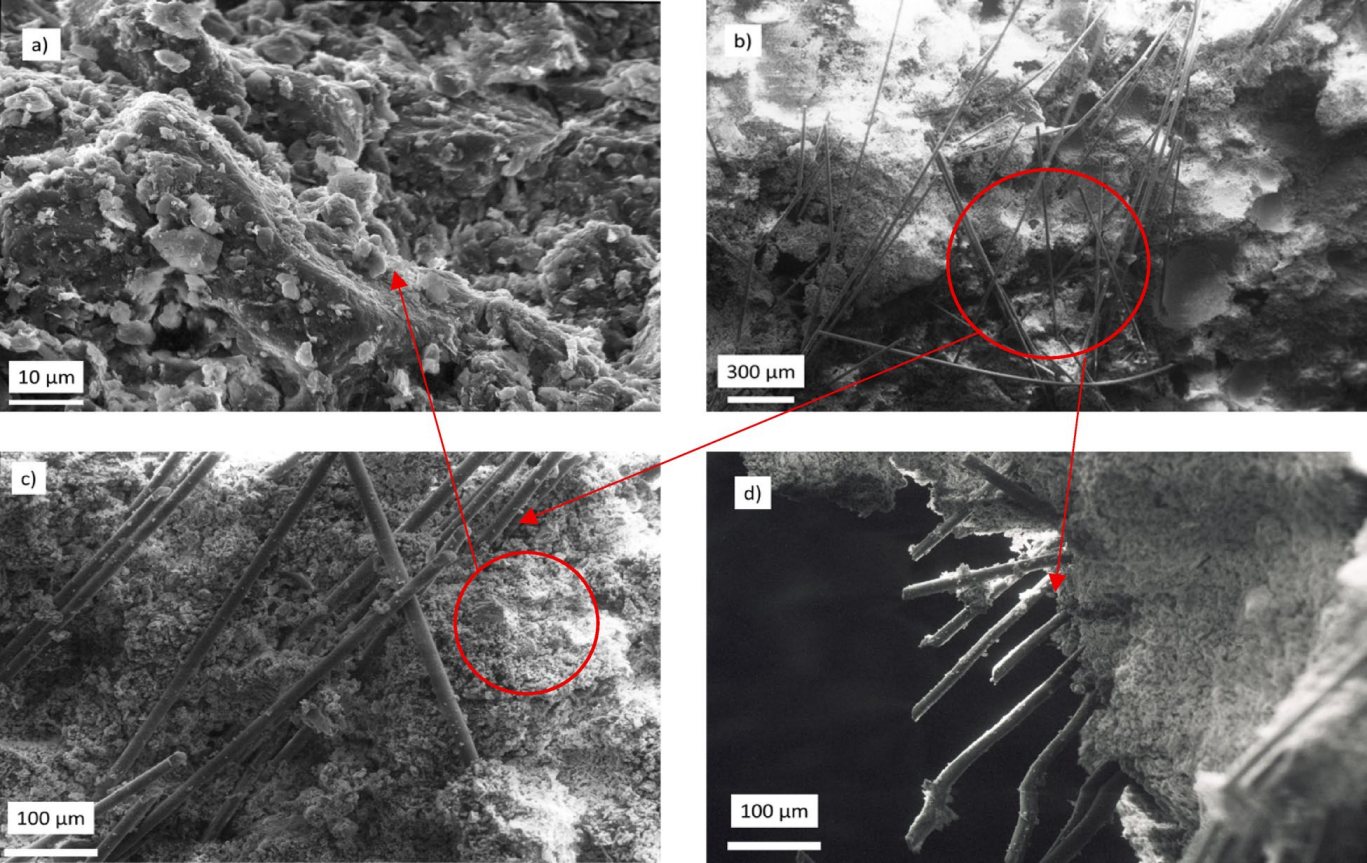
(**a**) Scanning Electron Microscopy (SEM) image showing the soil sample; (**b**), (**c**), and (**d**) SEM images of clay glass fiber highlighting random alignment and consistent distribution.

The microstructure of sample 2 is studied using scanning electron microscopy (SEM), as shown in Fig. 7. At low magnifications (Fig. 7a), the soil fabric exhibits a tightly packed matrix characterized by a developed network of clay particles alongside occasional larger silt grains. Higher magnification images (Fig. 7b and d) revealed the essential details of clay-coated glass fibers present within the matrix, which appear as a rod structure. These fibers are randomly oriented and well distributed, without significant agglomeration or void formation. The fibers exhibit a strong adhesion to the adjacent clay particles, suggesting direct effective stress transfer along the fiber matrix interface. These microstructural characteristics are likely to enhance the soil’s fracture resistance, facilitating processes such as crack bridging, crack tip shielding, and crack deflection, all of which are crucial for improving the toughness of materials. Chemical composition analysis through X-ray fluorescence (XRF), as shown in Table 2, further validates the choice of Sample 2 of CL soil.

(XRF).

**Table 2:** The elemental composition of the soil sample 2 derived from X-ray fluorescence.

Compound	SiO₂	Al₂O₃	Fe₂O₃	MgO	MnO	CaO	Na₂O	K₂O	TiO₂	*P*₂O₅	SO₃	Organic content
Value (%)	52.4	16.85	5.1	2.2	0.04	6.3	1.05	1.8	0.6	0.12	0.2	4.8

### Sample Preparation

Soil specimen for fracture toughness testing is prepared with meticulous attention to methodological consistency and specimen integrity. Each specimen is prepared with a width (*W*) and thickness (*B*) of 30 mm, a pre-crack length (*a*) of 10 mm, and an overall length of 100 mm. Initially, a specimen is created from low plasticity clayey soil (Sample 2) without any fiber reinforcement, and a baseline is formed for later comparisons. Glass fibers are then introduced into the soil at incremental weight fractions of 0.01%, 0.02%, 0.05%, and 0.10%, based on the dry weight of the soil. Before forming the specimens, the soil is prepared to achieve a density corresponding to the maximum dry density (MDD) determined from Standard Proctor compaction tests^120^. This step ensured consistency among all specimens and minimized variability due to density effects.

The mass of soil required for each specimen is calculated based on the volume of the cuboidal mould and the target dry density. The soil is uniformly mixed with the specified amount of glass fibers to ensure even distribution throughout the matrix. The mixture is then divided into three equal parts to allow for layered compaction within the mould. Each layer is sequentially placed into a cuboid (rectangular prism) box and compacted using a free-fall hammer system. The hammer, weighing approximately 500 g, is dropped freely onto the soil surface 25 times per layer to achieve uniform compaction and reduce the risk of internal voids. After compacting the first layer, the second and third portions of soil are sequentially added and compacted using the same procedure to complete specimen preparation. For each fiber–moisture condition, three replicate specimens were tested. The first specimen is prepared using a freshly mixed soil with optimum water content and compacted to the desired density. After testing, the same soil was gently disaggregated, rehydrated to maintain its original moisture content, and recompacted to produce the second specimen. The third specimen was prepared using the same procedure to ensure consistency in composition and compaction across all replicates. This approach allowed for controlled replication without introducing batch-to-batch variability, thereby enhancing the reliability of the mechanical response observed in the SENB tests. Special care is taken to maintain consistent compaction energy across all specimens. This is important because of the sensitivity of fracture toughness measurements to local density and material uniformity.

There is no specific ASTM standard currently established for the fracture testing of compacted soils. Therefore, sample preparation procedures are guided by established standards for related geotechnical tests. Soil compaction was performed in accordance with ASTM D698, and moisture conditioning and mixing followed procedures similar to those outlined in ASTM D4767 (triaxial compression). The prepared specimens were moulded in a cuboidal shape to accommodate the geometry required for SENB testing under a three-point bending configuration. The cuboidal shape enables precise notching, uniform support placement, and improved crack control during loading. This geometry is consistent with earlier studies on fracture behaviour in soils and soft rocks and aligns with the principles of ASTM E399, although adapted for compacted, unsaturated geomaterials.

After compaction, all specimens were carefully removed from the moulds and conditioned under controlled laboratory conditions to promote uniform moisture distribution before testing. The preparation process ensured the production of high-quality, reproducible samples suitable for evaluating fracture toughness, with a clear emphasis on minimizing experimental error and enhancing the reliability of test outcomes. Using a fixed specimen size may limit the generalizability of the results to field conditions, where stress distributions and boundary effects can vary significantly. Future studies may explore the influence of specimen geometry and scaling to capture size-dependent fracture behaviour in compacted soils.

### Fracture toughness testing

**Fig. 8:**
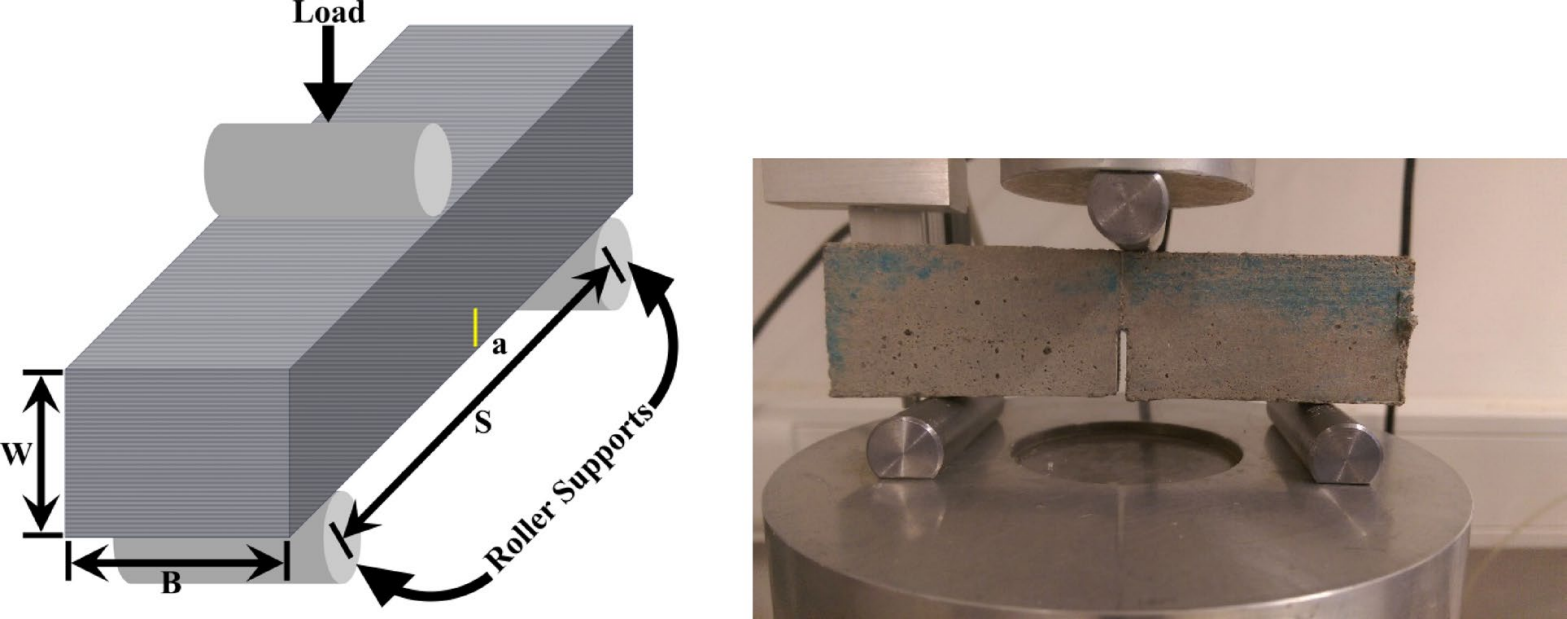
SENB test setup with a vertical load applied at the center, and beam dimensions (beam’s effective span is *S*, width is *W*, crack length is *a*, and thickness is *B*).

This investigation into fracture toughness involves SENB specimen made up of clayey soil reinforced with various percentages of glass fiber. As illustrated in Fig. 8, the dimensions of the specimen are carefully selected: a length (*L*) of 100 mm, a span (*S*) of 96 mm, a width (*W*) of 30 mm, and a thickness (*B*) of 30 mm, with an initial notch length (*a*) of 10 mm, serving as a pre-crack or stress concentrator. The sample is supported on rollers spaced at a distance *S* apart. The inclusion of glass fibers, measured as a percentage of the soil’s weight, is a strategic choice to improve the tensile strength and fracture resistance of the otherwise brittle clayey soil. During testing, the specimen undergoes three-point bending, where the applied load creates a stress concentration at the notch tip, encouraging stable crack growth until failure. This experimental arrangement allows for a consistent evaluation of the material’s fracture toughness, particularly highlighting the effect of fiber content on crack propagation resistance and energy absorption capacity.

The precision displacement sensor operates on the Linear Variable Differential Transformer (LVDT) principle, offering a measuring range of 0–25 mm with outstanding repeatability of better than 0.002 mm and linearity of 0.2% across the entire span. These features ensure highly reliable and accurate measurements, essential for research requiring utmost precision. The sensor supports a supply voltage of up to 42 V and a measuring frequency of 20 Hz, making it appropriate for static and moderately dynamic conditions. With a rugged design, it can function in a broad temperature range from −30 °C to + 100 °C. Although it has an IP 40 protection rating, its compact length of 65 mm allows for easy integration into experimental setups with limited space, attached to the vertical load cell. This sensor recorded vertical displacement at the crack mouth during SENB tests on clayey soil specimens. Initially, samples without glass fiber were prepared, followed by those with small percentages of glass fiber, as mentioned earlier. As shown in Fig. 8, the load was applied while the soil specimens were positioned on roller supports. Load and displacement data were gathered using the LVDT, and fracture toughness was calculated using Eqs. (1) and (2). The load-displacement responses showed minimal plastic deformation, affirming the near-elastic behaviour assumption. In line with established practices and ASTM guidelines, the maximum load, also known as the peak load *P*_max_, was used for fracture toughness calculations, mirroring methodologies applied to similar soils, such as clayey soils. Fracture in unsaturated soils is considered a thermodynamically irreversible process, contrasting with Griffith’s classical fracture theory for ideal brittle materials. In soils, the formation of new surfaces leads to additional energy dissipation mechanisms, including grain sliding, nonelastic deformation, and potential phase changes at crack surfaces. Only a portion of the energy contributes to breaking cohesive bonds. Consequently, the generalized term “resistance to fracture” represents the total work required to create new surfaces. The plastic zone at the crack tip remains minimal, allowing stress intensity factors to characterize the fracture process. To ensure valid plane strain conditions and precise toughness measurements, SENB samples are prepared with adequate thickness, maintaining an effective span-to-crack thickness ratio of approximately 10:1, as recommended. For elastic materials, fracture toughness was calculated using the equation proposed by Anderson and ASTM E399(Anderson, (2005) and ASTM E399-24, (2024) as shown below:

Fracture Toughness $${K_I}=\frac{P}{{B\sqrt W }}f\left( {\frac{a}{W}} \right)$$ (1).

where

2 $$f\left( {\frac{a}{W}} \right)=\frac{{3\left( {\frac{S}{W}} \right)\sqrt {\frac{a}{W}} }}{{2\left( {1+\left( {\frac{a}{W}} \right)} \right){{\left( {1 - \left( {\frac{a}{W}} \right)} \right)}^{1.5}}}}\left[ {1.99 - \frac{a}{W}\left( {1 - \frac{a}{W}} \right)\left\{ {2.15+3.93\left( {\frac{a}{W}} \right)+2.7{{\left( {\frac{a}{W}} \right)}^2}} \right\}} \right]$$

with.

$$0.45 \leqslant \frac{a}{W} \leqslant 0.55$$

In the above equations, *a* is the crack length, *W* is the width, *B* is the thickness, *S* is the spacing between roller supports, and *P* is the peak load.

**Fig. 9:**
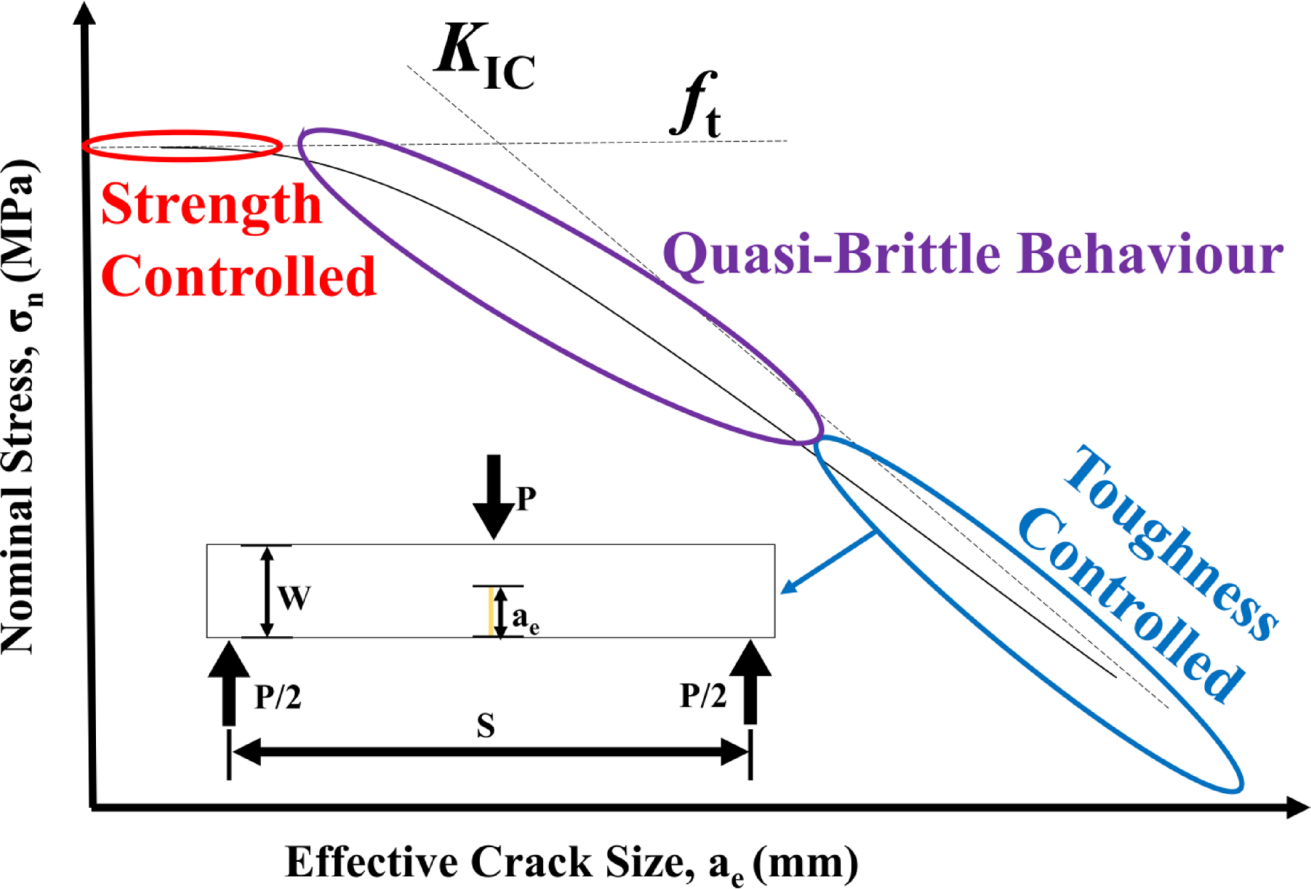
The correlation between nominal stress (σ_n_) and the effective crack size (a_e_) in a SENB test specimen.

Figure 9 illustrates the fundamental relationship between nominal stress (σₙ) and effective crack size (aₑ) in SENB testing, providing a basis for understanding material behaviour across various failure criteria. In the case of smaller cracks, the response is mainly strength-controlled; the material fails once the stress surpasses its intrinsic tensile strength, regardless of extensive crack growth. This consequence is typically associated with materials that have minimal or no stable cracks. As the crack size expands, the system shifts into a quasi-brittle behaviour, where strength and toughness factors compete, leading to some stable crack growth before catastrophic failure occurs. Ultimately, for larger cracks, the behaviour becomes toughness-controlled, where crack propagation takes precedence, and the material’s resistance to crack growth (fracture toughness) determines failure. In this phase, σₙ significantly diminishes as aₑ increases. The schematic in Fig. 9 illustrates the gradual shift from a strength-dominated failure mode to a toughness-dominated failure mode, highlighting the essential interaction between flaw size and material properties, which is important in designing and analyzing fiber-reinforced clayey soils for structural applications.

## Results and discussions

Results are presented to illustrate the influence of minimal and excessive fiber reinforcement on load-bearing capacity, energy absorption, and crack resistance. The discussion integrates experimental trends with fundamental concepts of unsaturated soil mechanics and fiber–soil interaction, providing insights into optimizing the design of fiber-reinforced clayey liners for improved durability and fracture resistance in landfill applications.

**Fig. 10:**
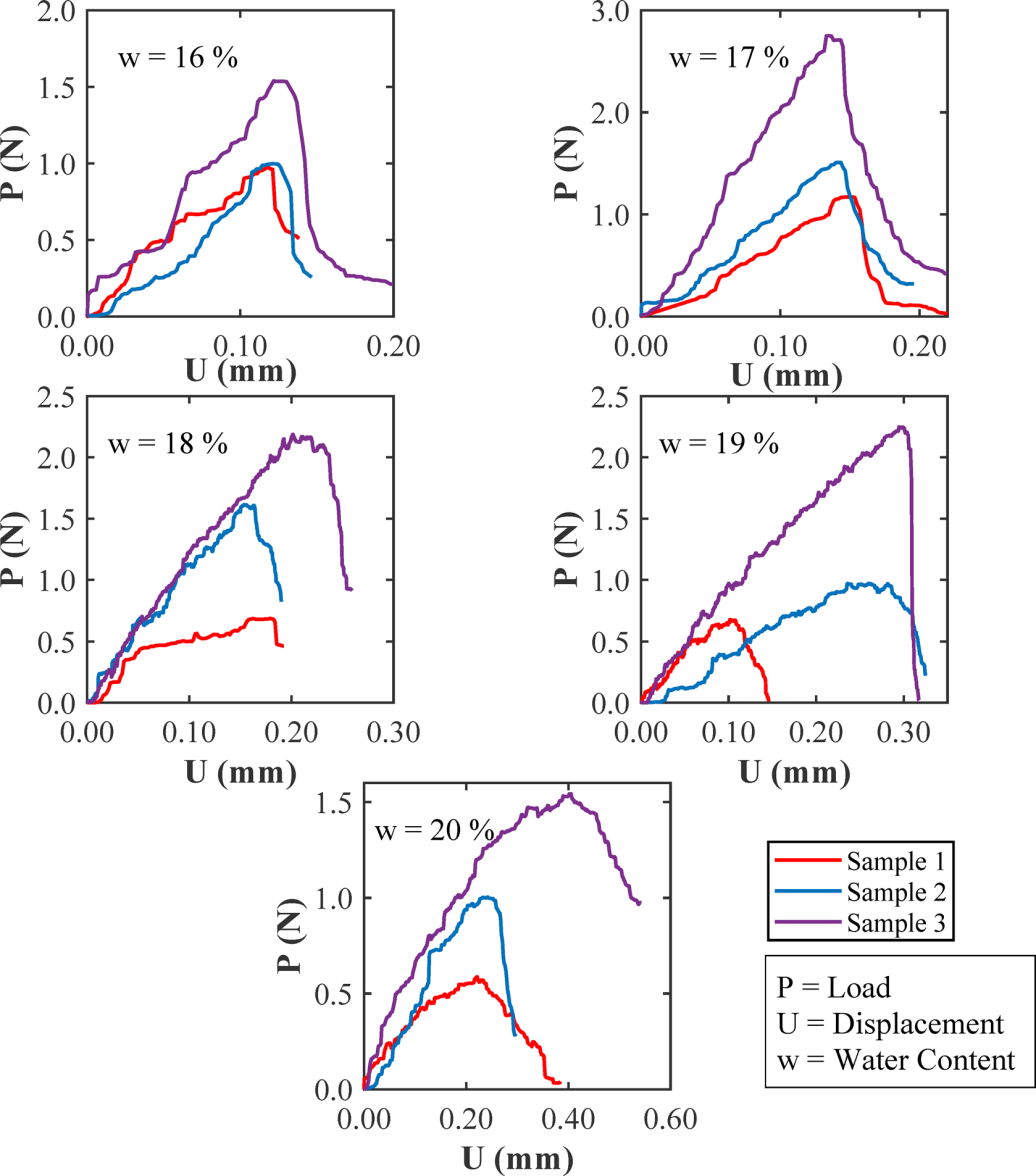
Load-displacement relationships from SENB fracture toughness tests without glass fibers.

Figure 10 shows the load (*P*) versus center-point displacement (*U*) curves from SENB fracture toughness tests on three clayey soil samples tested at different water contents (w = 16%, 17%, 18%, 19%, and 20%), without the addition of glass fibers. Since the samples had the same size and shape, the effect of water content on their fracture behaviour can be directly observed. At lower water contents of 16% and 17%, the samples (particularly Sample 3) reached higher peak loads (*P*_max_) and showed a sudden drop after that. This type of response is common in dry clays, where strong suction between particles makes the soil stiffer and more brittle. As water content increased to 18% and 19%, the shape of the curves changed. The load built up more gradually, and the drop after the peak was less drastic. This suggests that the soil became ‘soft’ and pliable. At 20% water content, the samples had even lower peak loads and could deform more before breaking. The load-displacement curves became wider, indicating that the soil could absorb more energy, although the strength continued to decline. Too much water acts like a lubricant, making the soil weaker and less stiff. The area under each curve shows how much energy the soil could absorb before failing. This area was largest for soils with medium water contents (around 17–19%). Dry soils are strong and brittle and breaks easily, while wet soils are plastic (undergoes more plastic deformation) and carry less load. The small differences between the three samples at each water content also show the natural variability in clay soils, especially when moisture and suction levels change. The variation in fracture toughness with water content is primarily governed by changes in matric suction and pore water distribution, as described by soil-water retention models such as those by Brooks–Corey and Fredlund–Xing^123^.

**Fig. 11:**
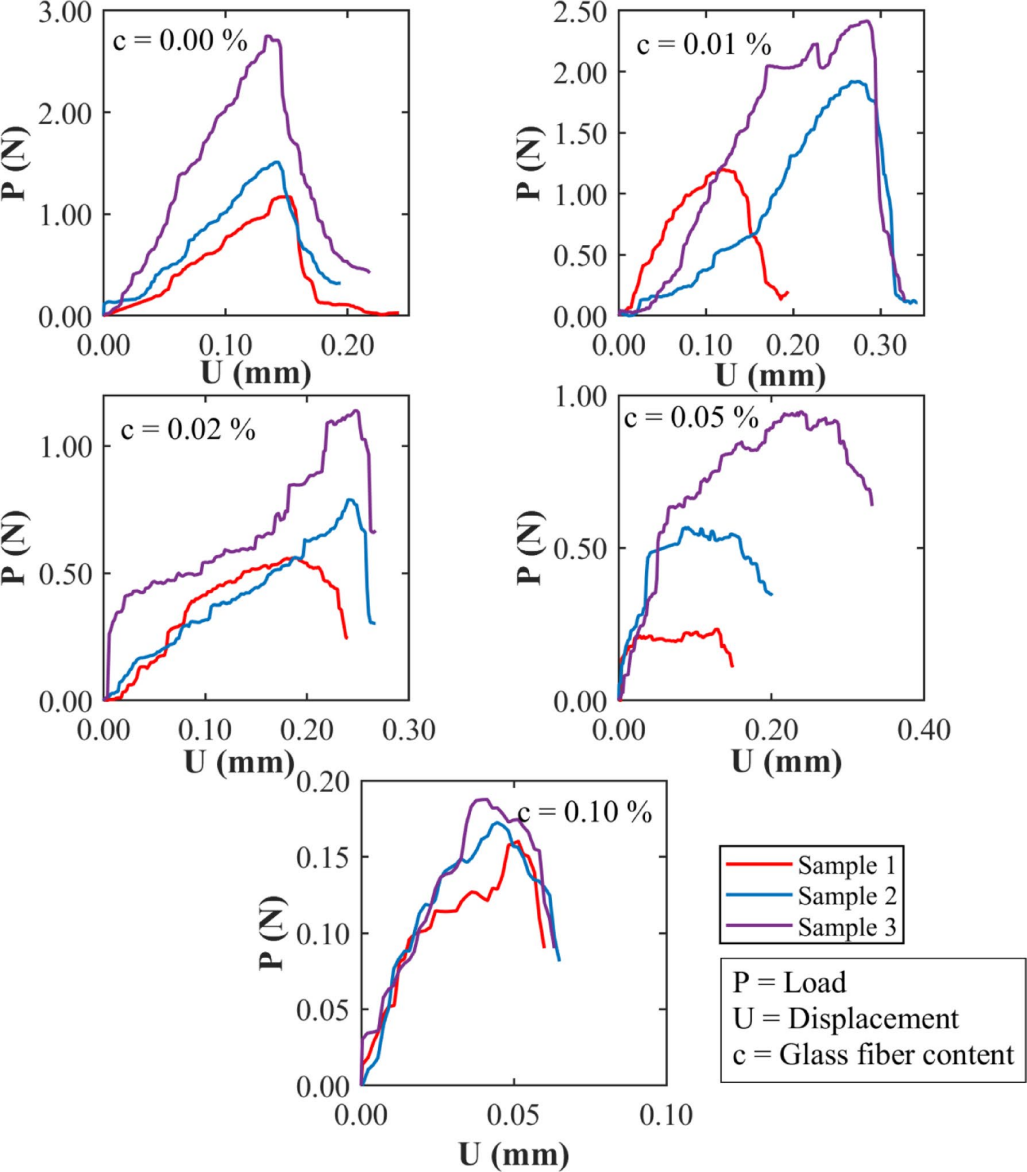
Load-displacement relationships of the SENB fracture toughness test at different percentages of glass fibers and at 17% water content (dry of optimum moisture content).

Figure 11 shows the load (*P*) versus center point displacement (*U*) curves of SENB fracture toughness tests on three clayey soil specimens with different glass fiber contents: 0.00%, 0.01%, 0.02%, 0.05%, and 0.10%. All samples were tested at a constant water content of 17%, which, as mentioned earlier, represents a relatively dry condition. Since the samples were all the same in shape and size, any difference in the response curves reflects mainly the effect of fiber content. For the soil without any fiber, the curves show a high peak load followed by a sharp drop, especially in Sample 3. This matches the brittle behaviour seen for unreinforced dry soil in Fig. 10. When small amounts of fiber are added, such as 0.01% and 0.02%, the curves become smoother, with several small drops along the way. This indicates that the fibers help control crack growth and improve the ability of the soil to absorb energy during loading. At 0.05% fiber content, the soil can hold a load over a larger displacement. This indicates better ductility and toughness, likely because the fibers are spread out well and work effectively to slow crack widening. But at 0.10%, the performance drops ࣧ the peak load is lower, and the curves soften earlier. This may be due to the presence of too much fiber clumping together and breaking the natural structure of the soil, thereby reducing its ability to carry loads. The area under the load displacement curve, which represents the energy the soil can absorb before failure, increases with fiber content up to around 0.02–0.05%. After that, it drops significantly. This means there is an optimal range within which the fibers improve performance, but beyond that point, they start to cause problems. Overall, the results show that adding small amounts of glass fibers can improve the behaviour of clayey soils under fracture loading, but excessive amounts can have the opposite effect.

**Fig. 12:**
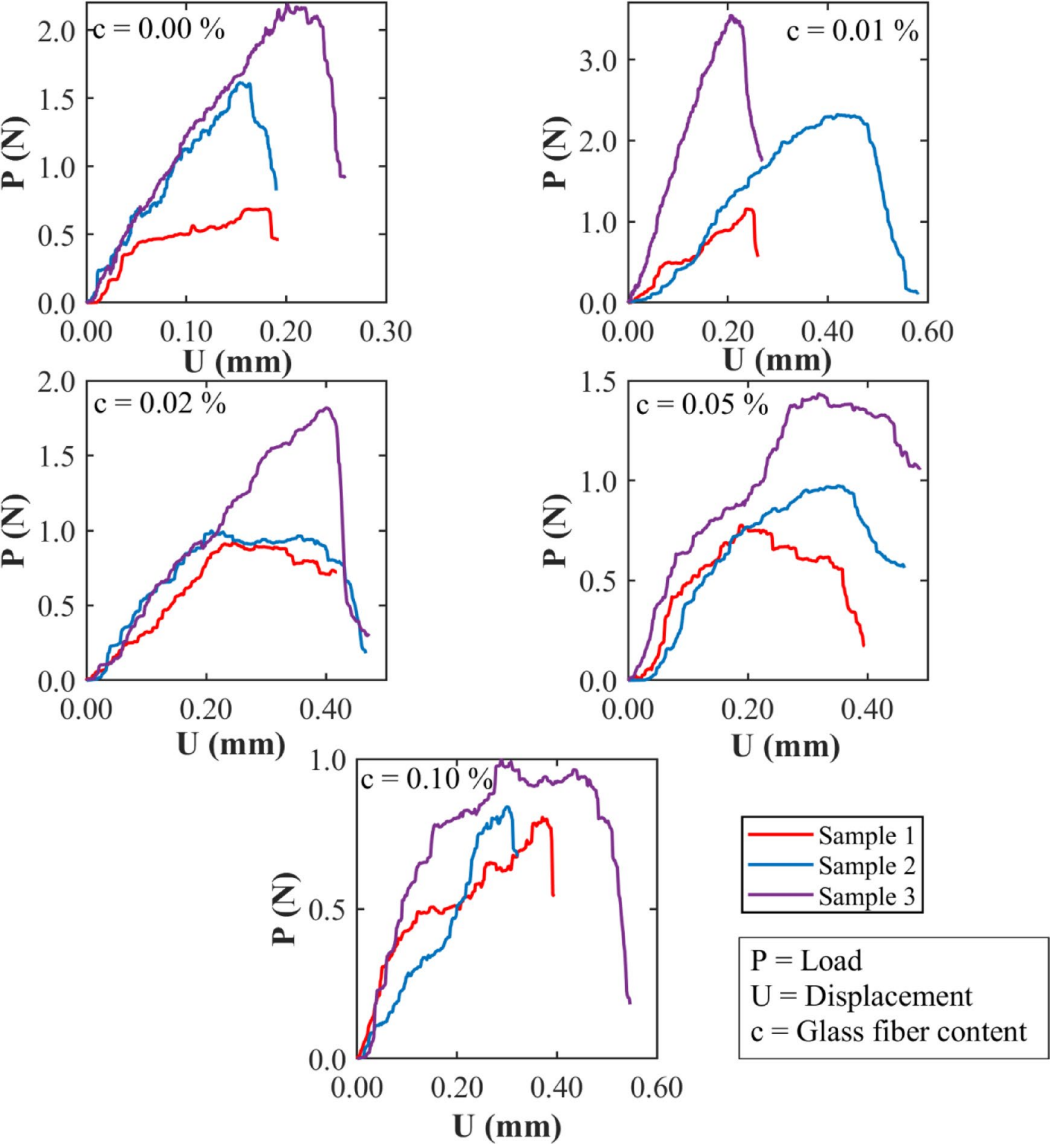
Load-displacement relationships of the SENB fracture toughness test specimens at different percentages of glass fibers and at 18% water content (vicinity of optimum moisture content).

Figure 12 presents the load (*P*) versus center point displacement (*U*) curves of SENB fracture toughness tests on three clayey soil specimens reinforced with glass fibers at 0.00%, 0.01%, 0.02%, 0.05%, and 0.10% fiber contents. The tests were conducted at a constant water content of 18%, which is close to the optimum moisture condition. The consistent sample geometry helps isolate the effect of fiber content under this moisture level. At 0.00% fiber content, the response shows moderate strength and limited displacement. As fibers are added, especially at concentrations of 0.01% and 0.02%, the curves become broader and more irregular, reflecting an increase in toughness. These changes continue at a rate of 0.05%, where the displacement before failure increases further, although the peak load remains relatively stable. At 0.10%, the curves flatten slightly and show a small drop in load-carrying capacity. This suggests that beyond a certain point, more fibers do not keep improving the behaviour and may even interfere with how the soil naturally holds together. The area under each curve increases with fiber content up to about 0.05%, then begins to level off or slightly decline. The results across the three samples show a consistent trend with minor differences likely due to natural variations. Figure 12 confirms that there is an optimal range of fiber content that improves soil performance at or near the optimum moisture, and adding more fibers than optimal may reduce the benefit.

**Fig. 13:**
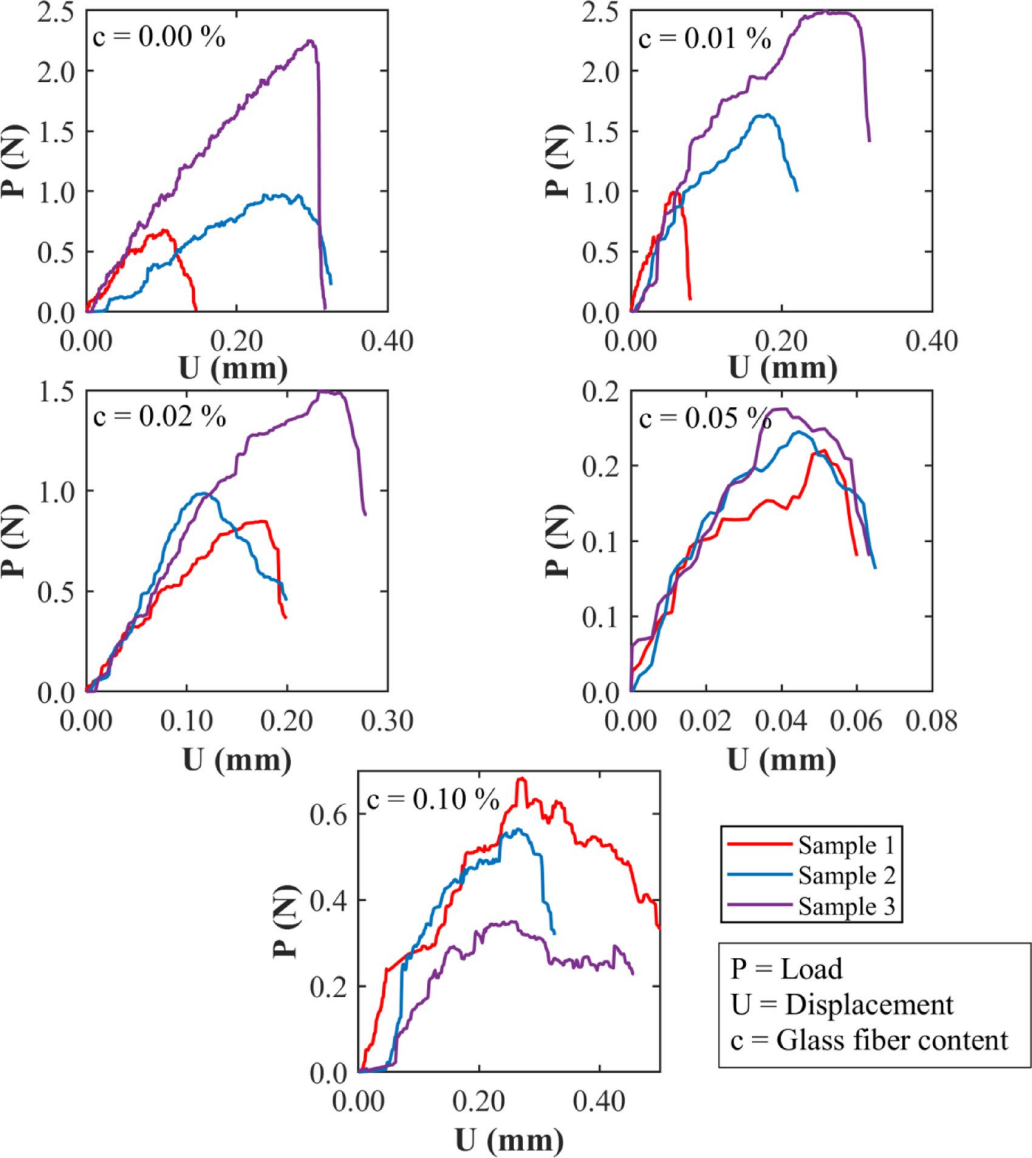
Load-displacement relationships of the SENB fracture toughness test specimens at different percentages of glass fibers and at 19% water content (wet of optimum moisture content).

Figure 13 shows the load (*P*) versus center point displacement (*U*) responses from SENB fracture toughness tests on clayey soil specimens with different glass fiber contents: 0.00%, 0.01%, 0.02%, 0.05%, and 0.10%. These tests were conducted at a water content of 19%, which is slightly greater than the soil’s optimum moisture level. Three samples were tested at each fiber level, and all the specimens had the same size and shape. At 0.00%, the curves rise quickly but then drop off sharply after reaching the peak load. This kind of response is expected in wetter clay where the soil becomes weaker and more pliable. When 0.01% of fibers are added, both the peak load and the post-failure displacement improve. The curves become wider, showing that the fibers are still helping the softer soil resist cracking. At 0.02%, this effect persists, with increased displacement before failure and greater energy absorption. However, at 0.05%, the curves flatten slightly, and the peak load starts to drop, suggesting that the higher moisture content makes it harder for the fibers to work effectively with the soil. When the fiber content is raised to 0.10%, the curves become uneven, and the strength drops further. The differences between the three samples also increase in size, which suggests uneven fiber distribution and poor bonding with the wet soil. The energy absorbed before fracture, reflected in the area under the curve, initially improves with fiber addition but begins to level off or decline at higher fiber contents. This shows that, while small amounts of fiber still help at this moisture level, adding too much fiber can reduce performance if the soil is already too wet. Figure 13 indicates that under wet conditions, only low to moderate fiber contents (0.01–0.02%) yield a meaningful improvement. This underscores the need for proper estimation of fiber content commensurate with the current moisture conditions in the field.

**Fig. 14:**
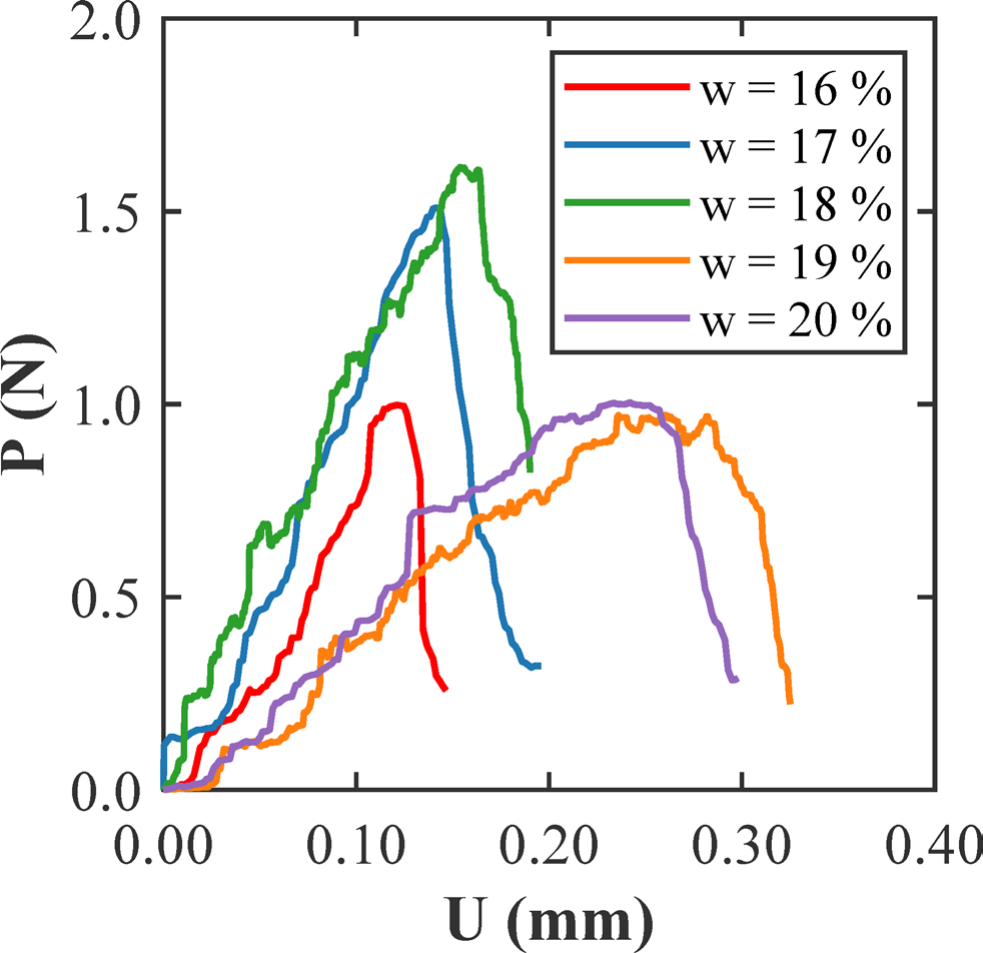
Load (P) – displacement (U) responses from the SENB fracture toughness tests on soil samples with different water contents (w) without glass fibers.

Figure 14 shows the load (*P*) versus displacement (*U*) behaviour of SENB fracture toughness tests on clayey soil samples without any glass fibers, tested at different water contents of 16%, 17%, 18%, 19%, and 20%. Each curve represents how the soil responds mechanically as the moisture level changes. At 16%, which is at the dry of optimum, the load rises quickly to a peak and then drops sharply. This kind of brittle response reflects a rigid soil behaviour with a limited ability to absorb fracture energy. As the water content increases to 17% and 18%, the curves broaden, and *P*_max_ increases. At 18%, which is close to the soil’s optimum moisture, the soil reaches its maximum strength. The improved load-bearing capacity at this point shows that the particles are better packed and more resistant to cracking. When the water content rises to 19% and 20%, the curves extend with lower peak loads but greater displacements before failure. This reflects a softer soil behaviour where excess water reduces internal friction, making it easier to deform and harder to carry heavy loads. Overall, the results in Fig. 14 indicate that moisture has a significant impact on the behaviour of clayey soils under stress. The soil performs best near its optimum moisture and becomes weaker and more flexible as it gets wetter. These observations underscore the importance of managing water content carefully when evaluating or utilizing clayey soils in structural applications.

**Fig. 15:**
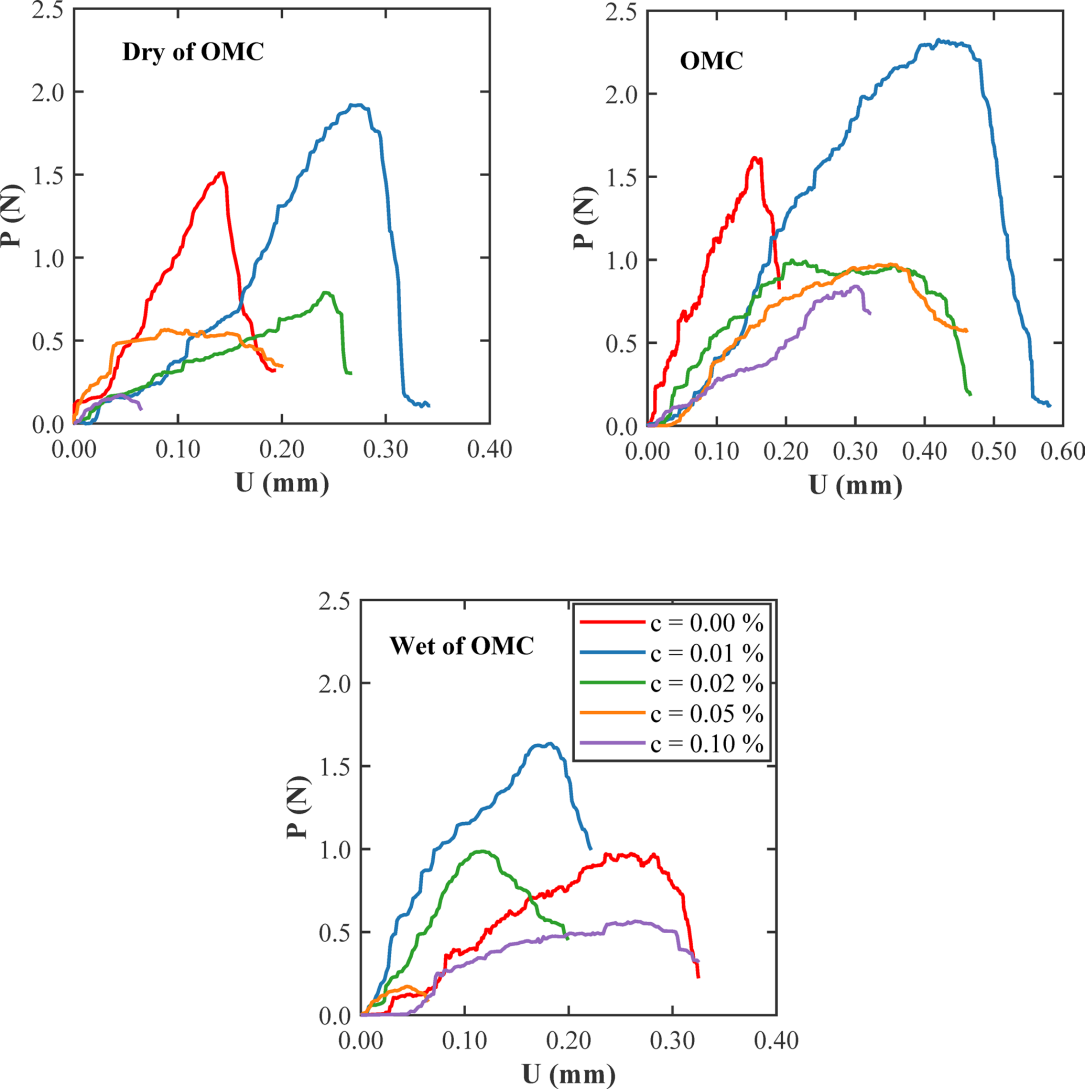
Load (P) - displacement (U) relationships from the SENB fracture toughness tests on soil samples with different percentages of glass fibers (c) and with (a) dry of optimum (17%), (b) optimum (18%), and (c) wet of optimum (19%) moisture contents (w).

Figure 15 compares the load (*P*) versus displacement (*U*) responses from SENB tests on clayey soil with increasing glass fiber content (c = 0.00%, 0.01%, 0.02%, 0.05%, and 0.10%) at three different water contents: 17% (dry of optimum), 18% (near optimum), and 19% (wet of optimum). In all three conditions, the unreinforced soil exhibits a brittle pattern, characterized by a moderate peak load followed by a sharp drop. The addition of 0.01% fibers consistently results in the most noticeable improvement. At 17% water content, the load-displacement curve becomes broader, and the peak load rises indicating that, even in drier soils, a small amount of fiber helps to bridge cracks and delay failure. As fiber content increases beyond 0.01%, the improvements decrease, with the curves showing reduced peak loads and smaller displacements, likely due to fiber clumping and limited bonding in the stiffer clay matrix. At 18%, which is near optimum, the performance improves overall, with the 0.01% fiber sample again showing the highest peak and widest curve. This reflects a good balance between fiber distribution and moisture conditions, allowing better energy absorption and stress redistribution. Samples with high fiber content at this moisture level exhibit a decrease in peak loads and do not provide any additional benefit. At 19% water content, where the soil is wet, the overall strength drops, but 0.01% fiber still leads to the most ductile response. Figure 15 highlights that a small fiber addition, especially around 0.01%, consistently offers the best improvement in strength and ductility across different moisture levels. This amount allows the fibers to work efficiently without interfering with the natural structure of the soil. At the same time, the water content continues to play a key role in setting the baseline behaviour.

**Fig. 16:**
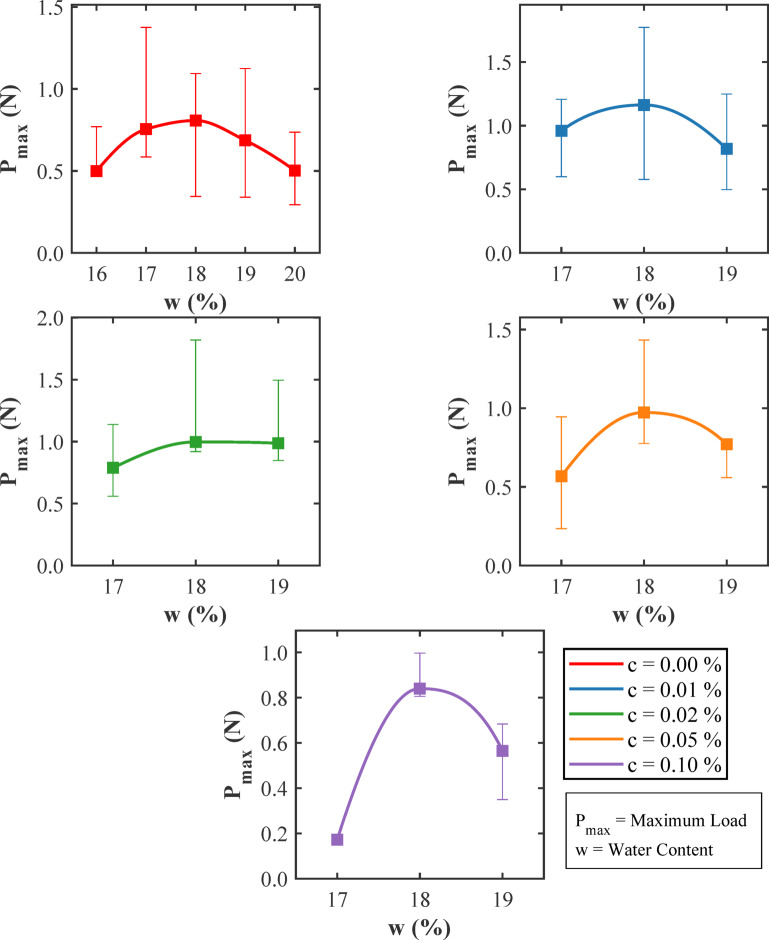
Variation of maximum load (P_max_) for three soil samples with different water content (w) corresponding to different amounts of glass fiber (c).

Figure 16 illustrates how the peak load sustained by SENB specimens varies with water content for different fixed amounts of glass fiber. Tests were repeated three times for each condition, and all samples shared the same geometry. For soil without fibers (c = 0.00%), the peak load rises from approximately 1.0 N at a 16% water content to around 1.6 N at 18%, then falls back to roughly 1.1 N at 20% water content. This pattern reflects stronger particle contact near the optimum moisture level and softening as the soil’s moisture content increases significantly. With 0.01% fibers, the peak loads are significantly higher for all the samples and reach approximately 2.4 N at 18% water content. The enhancement remains noticeable even at 17% and 19%, showing that a small amount of fiber addition amplifies the soil’s inherent strength over a wider moisture range. Moderate fiber contents of 0.02% and 0.05% produce a nearly flat trend for soil samples with 17–19% water content. Here, the peak load stays close to 1.0 N and shows less change with moisture. This suggests that, beyond the lightest reinforcement, the fibers no longer boost the load capacity in the same way. At 0.10% fiber content, the peak loads are the lowest, rising from 0.15 N at 17% to 0.85 N at 18% water content before dropping to 0.55 N at 19% water content. The reduced performance can be attributed to fiber clustering and weaker bonding at high fiber contents. The area under each load-displacement curve, which reflects energy absorption, is high for light fiber reinforcement and decreases at high fiber contents. The close match between replicate samples under each condition shows that the results are consistent and reliable. Overall, these results underline that fracture resistance in compacted clay depends on a balance between moisture and fiber contents. A very small fiber addition can drastically boost strength, but excessive fiber contents can reduce the benefits by disrupting the soil fabric.

**Fig. 17:**
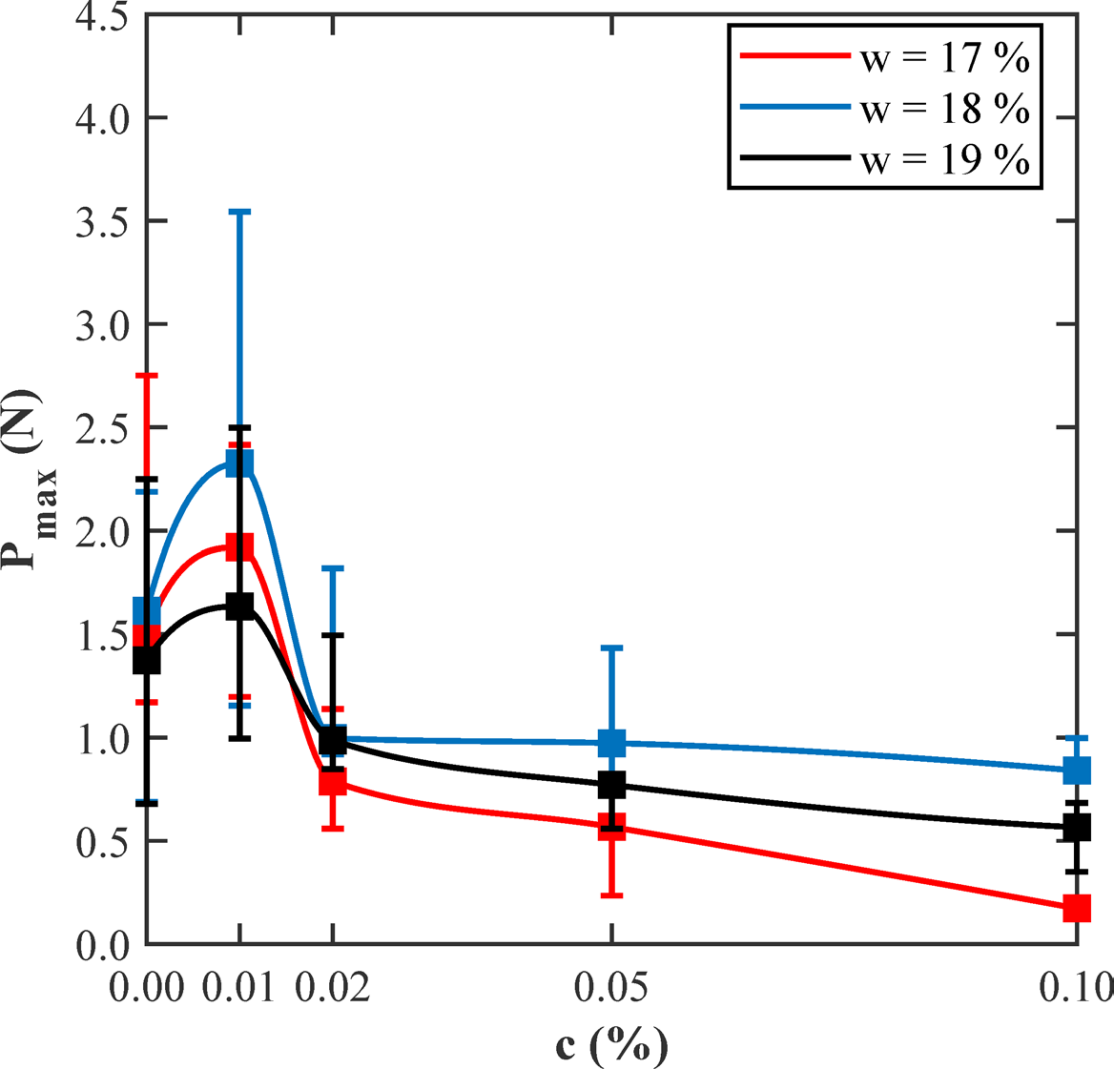
Variation of maximum load (P_max_) obtained from the SENB soil tests with different fiber content (c) at dry of optimum (17%), optimum (18%), and wet of optimum (19%) moisture contents (w).

Figure 17 shows the variation of peak load (*P*ₘₐₓ) observed in SENB tests with glass fiber content (c) for three distinct moisture levels: dry of optimum (17%), optimum (18%), and wet of optimum (19%). Each data point shown in the figure represents the average obtained from three replicate specimens, ensuring that comparisons between different fiber dosages and moisture conditions are consistent and reliable. Across all moisture levels, there is a clear and pronounced peak in the load sustained by the specimens at a very low fiber content, approximately 0.01%. At this optimal reinforcement level, the peak load reaches its highest values, ranging from about 2.3 to 2.4 N at the optimum moisture content (18%). This peak reflects the presence of an efficient fiber network that can bridge cracks effectively without disrupting the underlying soil structure or fabric. The fibers at this low concentration are well dispersed and interact synergistically with the soil matrix improving load transfer across developing fractures. When the fiber content increases beyond this 0.01% threshold, there occurs a sharp and consistent decline in the peak load values regardless of the moisture condition. At moderate fiber contents, ranging between 0.02% and 0.05%, peak loads reduce significantly to approximately 0.7 to 1.0 N. At the highest tested fiber content of 0.10%, the loads fall even further, reflecting the adverse effects of fiber clustering, formation of voids, and weakened bonding between the fibers and the soil matrix. This deterioration in mechanical performance occurs because excessive fiber additions compromise the soil’s packing density and matric suction, which are crucial for mechanical strength. The differences in peak load values across moisture contents highlight the critical role that matric suction and particle arrangement play in governing fracture resistance. Near the optimum moisture level, these factors are favourable for fiber-soil interactions, resulting in the highest peak loads. Conversely, drier (w = 17%) and wetter (w = 19%) conditions generally result in reduced peak loads demonstrating how moisture variations can adversely impact fracture toughness. In addition to peak load trends, the energy absorption capability of the specimens, which is indicated by the area under the load-displacement curves, also varies with fiber content and moisture level. At lower fiber dosages, the fiber reinforced soil absorbs more energy during fracturing, indicating that the material becomes tougher and fails in a ductile manner with gradual crack propagation. However, when fiber content increases beyond an optimal threshold, the material’s energy absorption capacity decreases, leading to brittle failure characterized by sudden, catastrophic fractures with sharp drops in load-carrying capacity immediately after peak stress. These observations highlight the importance of carefully balancing fiber dosage with soil moisture content to maximize the fracture resistance of fiber-reinforced clayey soils. Small fiber additions at or near optimum moisture content provides the best reinforcement effects, while excessive fiber contents can undermine soil integrity and mechanical performance.

**Fig. 18:**
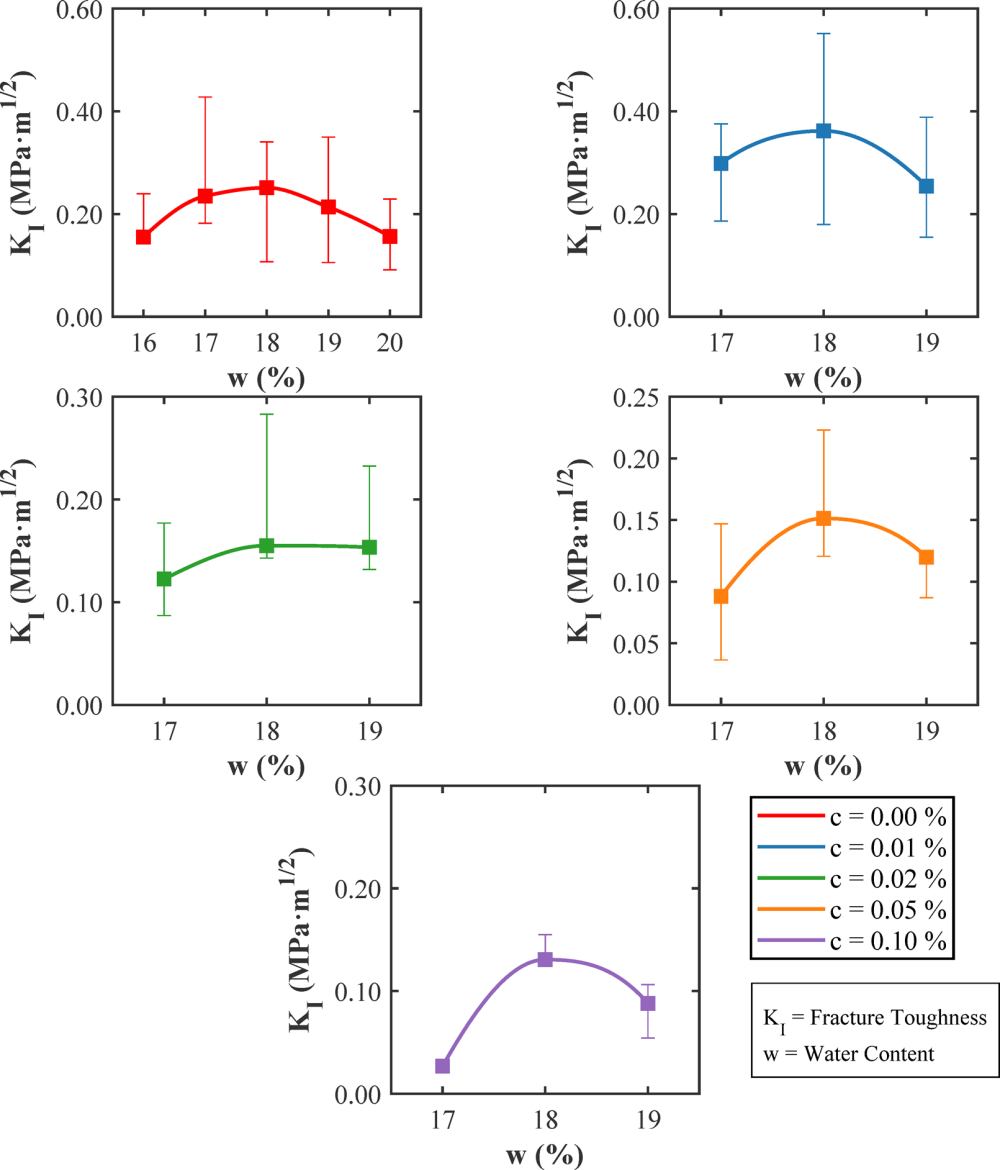
Variation of maximum fracture toughness (K_I_) with moisture content (w) levels for different amounts of glass fiber (c).

Figure 18 shows the variation of the critical stress intensity factor, commonly referred to as fracture toughness K_I_, with water content in clayey soils reinforced with varying amounts of glass fibers. The data came from three replicate SENB tests for dry of optimum, optimum and wet of optimum moisture conditions. As seen in earlier figures, fracture toughness reaches its maximum values near the soil’s optimum moisture content of approximately 18%, confirming the critical role of matric suction and particle arrangement on crack resistance, as discussed previously. For unreinforced soils, the fracture toughness begins low at a 16% water content, increases to a peak at the optimum moisture, and then declines as the water content rises further, illustrating the soil’s moisture-dependent strength characteristics explained in earlier sections. When a small quantity of glass fibers, specifically about 0.01%, is added, the fracture toughness improves significantly across all moisture levels, with notable maximums near the optimum moisture content. This improvement is consistent with the mechanisms of fiber bridging and crack tip blunting introduced before, which work most effectively at low fiber contents. At moderate fiber dosages, such as 0.02% and 0.05%, the fracture toughness also exhibits a local peak near the optimum moisture level, but with less pronounced improvement compared to the smallest fiber fraction. This reduction in effectiveness can be attributed to fiber clustering and reduced stress transfer, as discussed earlier. At the highest fiber content tested, 0.10%, fracture toughness values are the lowest among reinforced soils and the toughness only improved slightly even at at the optimum moisture content. This decrease confirms that excessive fiber content leads to the formation of voids and weak fiber-soil bonding, as explained earlier. Moreover, the variations in the shape and area under the fracture toughness curves show the changes in the energy dissipation capacity of the soils. Larger areas correspond to improved fracture toughness and energy absorption at low fiber contents, while smaller areas at high fiber dosages indicate more brittle behaviour and less effective energy dissipation. This phenomenon is tied back to the intrinsic moisture sensitivity and microstructural characteristics of clayey soils, as well as to how fiber reinforcement interacts with these factors. Figure 18 bolsters the conclusion that the optimal fracture toughness in glass fiber reinforced clays is achieved by balancing fiber content with moisture conditions. Minimal fiber additions near optimum moisture deliver the best results. In contrast, higher fiber contents reduce the material’s natural resistance to cracking due to adverse effects on the soil structure and fiber distribution.

**Fig. 19:**
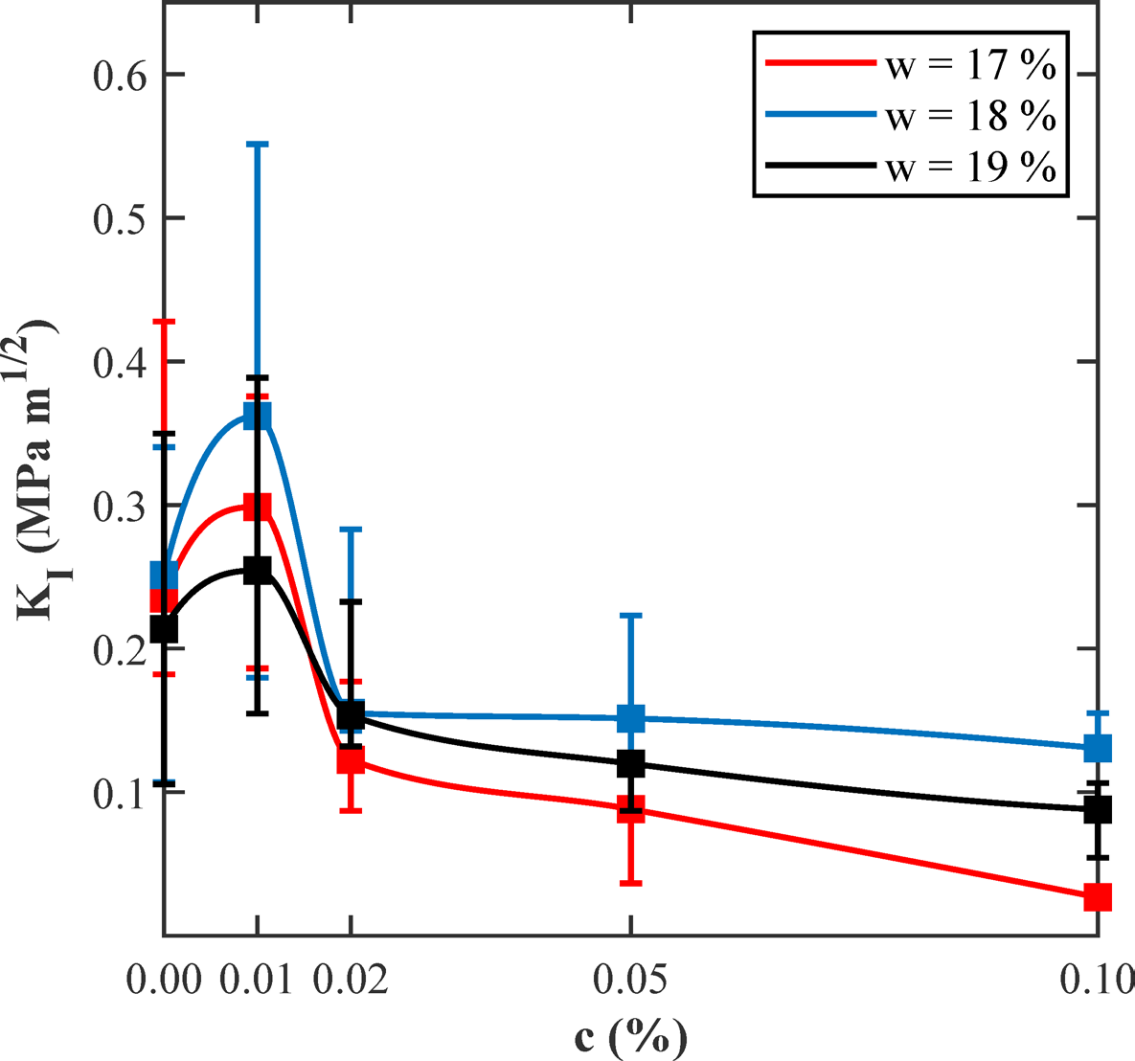
Variation of fracture toughness (K_I_) obtained from the SENB tests with glass fiber content (c) at dry of optimum (17%), optimum (18%), and wet of optimum (19%) moisture contents (w).

Figure 19 examines how fracture toughness, K_I_, varies with glass fiber content (c) in clayey soils tested under three moisture conditions (w = 17%, 18%, and 19%) using the SENB method. Load (*P*) and displacement (*U*) are recorded at the specimen’s center, with each value representing an average from three separate tests to ensure reliability. The data clearly show that the fracture toughness reaches its highest point at approximately 0.01% fiber content regardless of the moisture content. This suggests that there is an optimal fiber dosage at which reinforcement is most effective before adverse effects begin. At the optimum water content (18%), the soil-fiber composite achieves its peak toughness of 0.38 MPa·m^0^^5^ at this critical fiber content, underscoring a synergistic interaction between the fibers and soil matrix that effectively resists crack growth. However, increasing fiber content beyond this optimal dosage causes the toughness to steadily decline. This downward trend at higher fiber fractions can be attributed to fiber agglomeration and poor dispersion, which introduce flaws such as voids and weaken the mechanical cohesion within the matrix. Such structural irregularities hinder the transmission of stresses across cracks and lead to earlier material failures. Moisture content distinctly influences the magnitude and range of toughness variation for a particular fiber content. At 17% and 19% water contents, the toughness values are uniformly lower, indicating that deviations from the optimum moisture content restrict the capacity of the fiber-reinforced soil to resist fracture. The varying curve areas also reflect differences in the total energy the soil can absorb before fracturing under different moisture states, thus highlighting the role of water in facilitating or limiting the effectiveness of the presence of fibers. The data confirms that, in clayey soils, the delicate balance between fiber dosage and soil moisture is paramount. Excessive fiber addition does not improve toughness, rather undermines the matrix structure leading to brittleness and a loss of reinforcement benefits. The findings bolster the concept that successful fiber reinforcement is not simply a matter of increasing fiber content, rather requires precise control commensurate with soil moisture conditions. The narrow optimal window for fiber content and water level seen here highlights the need for tailored approaches in fiber-reinforced soil engineering to maximize fracture toughness without compromising integrity.

## Conclusions

In this study, the authors have established that moisture content and glass-fiber reinforcement significantly influence the fracture behaviour of compacted clay liners. All water content levels tested remained below saturation, which allowed capillary suction to dictate the matrix cohesion. The introduction of fiber reinforcement effectively enhanced the fracture toughness, leading to several critical findings:

The fracture resistance of soil exhibits a strong moisture dependence with both P_max_ and K_I_ reaching their maximum values at the soil’s optimum compaction moisture content of approximately 18%. Matric suction and particle interlocking are optimized at this point, enhancing the soil’s mechanical performance.

Optimal fiber dosage: A minimum glass-fiber content of approximately 0.01% by mass yields substantial enhancements in *P*_max_ (up to 50%) and K_I_ (up to 70%). These improvements are consistent across different soil moisture contents including dry of optimum, optimum, and slightly wet of optimum.

Adverse impacts of excessive reinforcement: Fiber contents equal to or exceeding 0.02% can disrupt the clay matrix by inducing clustering and creating voids, thereby compromising the integrity of soil-fiber bonding. This interference ultimately results in reduced strength and a tendency towards more brittle failure in the material.

The energy dissipation characteristics, indicated by the area under the load-displacement curves, peak at optimal moisture levels with minimal fiber content. However, this fracture energy absorption diminishes significantly when the soil conditions are excessively dry, too saturated, or when there is an overabundance of reinforcement.

Design considerations for landfill liners: Compacting soils at or near their OMC is important for ensuring the durability and crack resistance of clay liners. In instances where reinforcing fibers are incorporated, their addition should be restricted to small amounts, approximately 0.01%. Excessive presence of moisture or over-reinforcement can significantly undermine the integrity of the liner system.

By effectively aligning moisture management with strategically dispersed fiber reinforcement, engineers can decisively leverage clay’s natural cohesion (matric suction) and fibers’ crack-bridging properties to greatly enhance the durability and reliability of landfill containment systems.

Future research can build upon the findings of this study by integrating advanced numerical modelling techniques, such as finite element and discrete lattice element methods, to simulate the propagation of fractures and interactions between moisture and suction in fiber-reinforced clayey soils. These models can provide a mechanistic understanding of the crack-bridging and pull-out behaviour observed in the experiments. Additionally, long-term performance evaluations under cyclic wetting and drying conditions, as well as environmental stressors, could provide insights into the durability of the reinforcement over time. Exploring other types of discrete and/or natural fibers, along with hybrid reinforcement strategies, may further optimize fracture resistance while maintaining sustainability and cost-effectiveness. Application of the developed methodologies to field-scale testing and large-scale numerical modelling of crack propagation in landfill liners should help assess the scalability and practical relevance of the laboratory findings. Finally, scaling the current laboratory procedures to field-representative conditions would be valuable for validating the applicability of these findings in real landfill liner systems.

## Data Availability

No datasets were generated or analysed during the current study.

## Abbreviations

K_I_: Mode I fracture toughness
P: Load at the time of data recording
P_max_: Maximum / Peak load
OMC: Optimum moisture content
SENB: Single edged notched bend
MDD: Maximum dry unit weight
w: Water content
c: Glass fiber content
U: Displacement
σ_t_: Tensile strength
aₑ: Effective crack size
